# Supplementary material for: Natural Product-Derived Phosphonic Acids as Corrosion Inhibitors for Iron and Steel
Source: Molecules. 2022 Mar 8;27(6):1778. doi: 10.3390/molecules27061778 (PMC8955919; doi:10.3390/molecules27061778)
Supplement: Supplementary file 1 [file molecules-27-01778-s001.zip › molecules-1624450-supplementary.pdf]

# Supporting Information

## Natural Product-Derived Phosphonic Acids as Corrosion Inhibitors for Iron and Steel

Erik Ruf [a], Tim Naundorf [a], Tom Seddig [a], Helmut Kipphardt [b] und Wolfgang Maison *,[a]

[a]*Department of Chemistry, Universität Hamburg, Bundesstraße 45, 20146 Hamburg, Germany*

[b]*Metall-Chemie Technologies GmbH, Kaiser-Wilhelm-Straße 93, 20355 Hamburg, Germany*

*maison@chemie.uni-hamburg.de*

# Table of contents

Synthesis ... 1
Palladium-catalyzed hydrophosphinylation (general procedure) ... 1
10-Carboxyundecyl phosphonic acid 3[1] ... 1
(*E*)-(3,7-dimethylocta-2,6-dien-1-yl)phosphonic acid 6[2] ... 2
((3,3-Dimethylbicyclo[2.2.1]heptan-2-yl)methyl)phosphonic acid 7 ... 2
(2-(2-hydroxy-4-methylcyclohexyl)propyl)phosphonic acid 8 ... 3
((6,6-Dimethylbicyclo[3.1.1]heptan-2-yl)methyl)phosphonic acid 9 ... 3
((4-Isopropylcyclohex-1-en-1-yl)methyl)phosphonic acid 12[3] ... 4
(2-Methyl-5-(1-phosphonopropan-2-yl)cyclohexyl)phosphonic acid 14 ... 5
((4-Isopropyl-2-phosphonocyclohexyl)methyl)phosphonic acid 15 ... 6
(4,4a-Dimethyl-6-(1-phosphonopropan-2-yl)decahydronaphthalen-1-yl)phosphonic acid 16 ... 7
10-Phosphonooctadecanoic acid 18[5] ... 8
10-(Diethoxyphosphoryl)octadec-9-enoic acid 23 ... 9
10-Phosphonooctadec-9-enoic acid 24 ... 10
Diethyl (3,7-dimethyloct-6-en-1-yl)phosphonate 26 ... 11
(3,7-Dimethyloct-6-en-1-yl)phosphonic acid 27 ... 12
(3,7-Dimethyloctyl)phosphonic acid 28 ... 12
CMC Evaluations ... 14
Experimental procedure ... 14
Measured CMCs (Table S1) ... 14
NMR Spectra ... 15
NMR-Spectra ... 20
10-Carboxyundecyl phosphonic acid 3 ... 20
(E)-(3,7-dimethylocta-2,6-dien-1-yl)phosphonic acid 6 ... 22
((3,3-Dimethylbicyclo[2.2.1]heptan-2-yl)methyl)phosphonic acid 7 ... 23
(2-(2-hydroxy-4-methylcyclohexyl)propyl)phosphonic acid 8 ... 25
((6,6-Dimethylbicyclo[3.1.1]heptan2-yl)methyl)phosphonic acid 9 ... 27
((4-Isopropylcyclohex-1-en-1-yl)methyl)phosphonic acid 12 ... 29
(5-(1-(diethoxyphosphoryl)propan-2-yl)-2-methylcyclohexyl)phosphonate ... 31
(2-Methyl-5-(1-phosphonopropan-2-yl)cyclohexyl)phosphonic acid 14 ... 32
Diethyl ((2-(diethoxyphosphoryl)-4-isopropylcyclohexyl)methyl)phosphonate ... 33
((4-Isopropyl-2-phosphonocyclohexyl)methyl)phosphonic acid 15 ... 34
(6-(1-(diethoxyphosphoryl)propan-2-yl)-4,4a-dimethyldecahydronaphthalen-1-yl)phosphonate ... 35

(4,4a-Dimethyl-6-(1-phosphonopropan-2-yl)decahydronaphthalen-1-yl)phosphonic acid 16 ........ 36
10-(diethoxyphosphoryl)octadecanoic acid ........ 37
10-Phosphonooctadecanoic acid 18 ........ 39
10-(Diethoxyphosphoryl)octadec-9-enoic acid 23 ........ 41
10-Phosphonooctadec-9-enoic acid 24 ........ 42
Diethyl (3,7-dimethyloct-6-en-1-yl)phosphonate 26 ........ 43
(3,7-Dimethyloct-6-en-1-yl)phosphonic acid 27 ........ 45
Diethyl (3,7-dimethyloctyl)phosphonate ........ 47
(3,7-Dimethyloctyl)phosphonic acid 28 ........ 49
Chip filter test (Table S2) ........ 51
Nyquist Plots ........ 52
References ........ 52

# Synthesis

**Palladium-catalyzed hydrophosphinylation (general procedure)**

$Pd_2dba_3$ (75%, 0.01 eq., 0.1 mmol, 122 mg) and Xantphos (0.022 eq., 0.22 mmol, 127 mg) were dissolved in 10 mL of DMF. Hypophosphorous acid (50% in $H_2O$, 2 eq., 20 mmol, 2.35 mL) and the olefin, or the allylic alcohol (1 eq., 10 mmol) were added. The reaction mixture was heated to 110° C for 16 h The reaction mixture was then filtered and the solvent evaporated under reduced pressure. The residue was dissolved in 50 mL of 2 M aqueous HCl and 50 mL of EtOAc. The layers were separated and the aqueous layer extracted three times with 50 mL of EtOAc. The combined organic layers were washed with 50 mL of saturated NaCl-solution and dried over $Na_2SO_4$. Removal of the solvent gave the crude product which was oxidized or purified *via* RP column chromatography as specified.

**10-Carboxyundecyl phosphonic acid 3**[1]

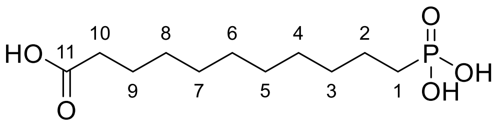


10-Undecylenic acid **1** (82.8 g, 450 mmol, 1.0 eq) was treated according to the general procedure. After 16 h, quantitative conversion to the intermediate phosphinic acid was detected via mass spectrometry. The solution was cooled to 0°C and 30% aqueous $H_2O_2$ (102 g, 900 mmol, 2 eq) was added over 120 min. The reaction mixture was then heated to 110° C for 60 min. The resulting mixture was filtered and the solvent was evaporated under reduced pressure. The residue was treated with 250 mL 2 M aqueous HCl and 250 mL of EtOAc. The organic layer was separated and the aqueous layer extracted three times with each 250 mL EtOAc. The combined organic layers were washed with 250 mL saturated NaCl-solution and dried over $Na_2SO_4$. After crystallisation from acetone and subsequent freeze drying 119.5 g (449 mmol, 99%) of the title compound **3** were obtained as a colorless solid.

$^1H$ NMR (600 MHz, MeOD-$d_4$) $\delta$ = 2.29 (dt, $^2J$ = 20.4 Hz, $^3J$ = 7.4 Hz, 2H, 10-H), 1.71 – 1.64 (m, 2H, 1-H), 1.60 (dqd, $^2J$ = 12.1 Hz, $^3J$ = 7.4, 6.6, 3.8 Hz, 4H, 3-H, 9-H), 1.41 (p, $^3J$ = 6.6 Hz, 2H, 2-H), 1.36 – 1.28 (m, 10H, 4-H, 5-H, 6-H, 7-H, 8-H).

$^{13}$C NMR (151 MHz, MeOD-$d_4$) $\delta$ = 177.65 (C11), 34.86 (d, $^2J_{P,C}$ = 23.2 Hz, C10), 31.75 ( d, $^2J_{P,C}$ = 16.6 Hz, C2), 30.51 (d, $^2J_{P,C}$ = 3.2 Hz, ($CH_2$)), 30.44 ($CH_2$), 30.35 (d, $^2J_{P,C}$ = 6.0 Hz, ($CH_2$)), 30.23 (d, $^2J_{P,C}$ = 8.7 Hz, ($CH_2$)), 30.15 ($CH_2$), 28.09 (d, $^2J_{P,C}$ = 137.9 Hz, C1), 26.03 (d, $^2J_{P,C}$ = 10.0 Hz, C9), 23.91 (d, $^2J_{P,C}$ = 5.0 Hz, C3).

$^{31}$P NMR (243 MHz, MeOD-$d_4$) $\delta$ = 30.34.

HRMS (ESI) m/z: [M-H]$^-$ calculated for $C_{11}H_{22}O_5P$: 265.1210, found 265.1210.

**(*E*)-(3,7-dimethylocta-2,6-dien-1-yl)phosphonic acid 6**[2]

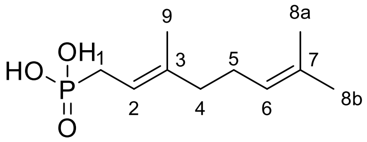


1 eq., 10.0 mmol, 1.75 mL) was treated according to the general procedure. ($H_2O$:MeCN 95:5 V./V. → 5:95 V./V., 0.1% HCOOH). Freeze drying gave 1.29 g (5.91 mmol, 60%) of the title compound **6** as colorless solid.

**((3,3-Dimethylbicyclo[2.2.1]heptan-2-yl)methyl)phosphonic acid 7**

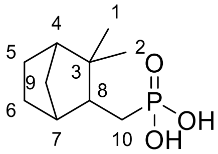


Camphene (272 mg, 2.00 mmol, 1.0 eq) was treated according to the general procedure. T($H_2O$:MeCN 50:1 V./V. → 1:50 V./V., 0.1% HCOOH). Freeze drying gave 200 mg (91.8 µmol, 46%) of the title compound **7** as a colorless solid.

$^1$H NMR (600 MHz, $CDCl_3$) $\delta$ = 10.34 (s, 2H, O-H), 2.33 (s, 0.8H, $7_a$-H), 2.23 (s, 0.2H, $7_b$-H) 1.88 – 1.78 (m, 1H, 4-H), 1.77 (s, 1H, 8-H), 1.76 – 1.69 (m, 2H, 9-H), 1.67 – 1.62 (m, 1H, $10_a$-H), 1.57 – 1.51 (m, 1H, $5_a$-H), 1.34 – 1.28 (m, 2H, 6-H), 1.28 – 1.22 (m, 1H, $10_b$-H), 1.18 (dt, $J$ = 9.8, 1.7 Hz, 1H, $5_b$-H), 1.00 (s, 0.5H, $1_a$-H), 0.96 (s, 2.5H, $1_b$-H), 0.88 (s, 0.5H, $2_a$-H), 0.80 (s, 2.5H, $2_b$-H).

$^{13}$C NMR (151 MHz, $CDCl_3$) $\delta$ = 48.74 (C8), 44.19 (C4), 44.05 ($C7_a$), 42.30 ($C7_b$), 37.63 (d, $^2J_{P,C}$ = 14.7 Hz, C3), 37.09 (C10), 31.86 ($C1_a$), 29.58 ($C5_a$), 27.52 ($C1_b$), 25.50 ($C2_b$), 24.71 (C5), 24.20 ($C9_a$), 23.40 ($C9_b$), 22.03 ($C2_a$), 20.25 (C6).

$^{31}$P NMR (243 MHz, $CDCl_3$) $\delta$ = 38.34, 38.07.

HRMS (ESI) m/z: $[M-H]^-$ calculated for $C_{10}H_{19}O_3P$: 217.0999, found 217.0999.

**(2-(2-hydroxy-4-methylcyclohexyl)propyl)phosphonic acid 8**

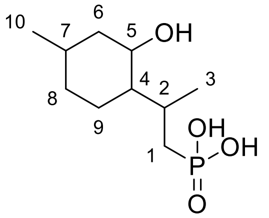


Isopulegol (308 mg, 2.00 mmol, 1.0 eq) was treated according to the general procedure. T($H_2O$:MeCN 50:1 V./V. → 1:50 V./V., 0.1% HCOOH). Freeze drying gave 102 mg (43.2 µmol, 22%, isolated isomer: 23 mg, 9.73 µmol, 5%) of the title compound **8** as a colorless solid.

$^1$H NMR (600 MHz, MeOD-$d_4$) $\delta$ = 4.19 (ddt, $^3J$ = 10.7, 5.0, 1.7 Hz, 1H, 5-H), 2.30 – 2.17 (m, 1H, 2-H), 2.00 (ddd, $^2J$ = 12.3 Hz, $^3J$ = 5.0, 3.5, Hz, 1H, $6_a$-H), 1.94 (dd, $^2J$ = 14.8 Hz, $^3J$ = 6.1 Hz, 1H,$1_a$-H), 1.80 (dd, $^2J$ = 14.8 Hz, $^3J$ = 2.8 Hz, 1H, $1_b$-H), 1.71 – 1.65 (m, 1H, $9_a$-H), 1.58 – 1.50 (m, 2H,4-H, $8_a$-H), 1.44 (dddq, $^3J$ = 15.4, 11.9, 6.6, 3.5 Hz, 1H, 7-H), 1.32 – 1.23 (m, 1H, $8_b$-H), 1.19 – 1.14 (m, 1H, $6_b$-H), 1.13 (d, $^3J$ = 7.2 Hz, 3H, 3-H), 0.99 – 0.95 (m, 1H, $9_b$-H), 0.94 (d, $J$ = 6.6 Hz, 3H, 10-H).

$^{13}$C NMR (151 MHz, MeOD-$d_4$) $\delta$ = 77.66 (d, $^4J_{P,C}$ = 6.1 Hz, C5),45.84 (d, $^3J_{P,C}$ = 7.5 Hz C4), 43.71(C6), 35.41 (C9), 33.00 (d, $^2J_{P,C}$ = 6.7 Hz, C2), 32.23(C7), 31.68 (d, $^1J_{P,C}$ = 124.5 Hz, C1), 29.41 (C8), 22.32 (C10), 14.49 (C3).

$^{31}$P NMR (243 MHz, MeOD-$d_4$) $\delta$ = 23.66.

HRMS (ESI) m/z: $[M-H]^-$ calculated for $C_{10}H_{21}O_4P$: 235.1105, found 235.1095.

$[M-H_2O-H]^-$ calculated for $C_{10}H_{21}O_4P$: 217.0999, found 217.0990.

**((6,6-Dimethylbicyclo[3.1.1]heptan-2-yl)methyl)phosphonic acid 9**

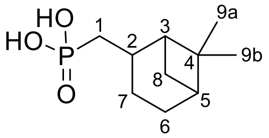

1 eq., 10.0 mmol, 1.57 mL) was treated according to the general procedure. ($H_2O$:MeCN 95:5 V./V. → 5:95 V./V., 0.1% formic acid). Freeze drying gave 240 mg (1.1 mmol, 11%) of the title compound **9** as colorless solid, containing ~ 15% of the ring opened derivative **12** according to $^1$H-NMR.

$^1$H NMR (300 MHz, MeOD) $\delta$ = 2.56 – 2.30 (m, 1 H, 2-H), 2.21 – 2.01 (m, 1 H, 7-Ha), 1.93 – 1.72 (m, 6 H, 3-H, 5-H, 6-H, 8-H), 1.71 – 1.59 (m, 2 HH, 9a-H), 0.88 (s, 3 H, 9b-H).

$^{13}$C NMR (100 MHz, MeOD, 25 °C) $\delta$ [ppm] = 48.4 (C5), 41.9 (C3), 35.3 (d, $^1J$ = 135.8 Hz, C1), 31.5 (d, $^2J$ = 3.8 Hz, C2), 27.2 (C9a), 25.5 (C4), 24.5 (C8), 24.4 (C6), 23.8 (C7), 20.4 (C9b).

$^{31}$P NMR (162 MHz, MeOD) $\delta$ = 29.46 (s).

HRMS-ESI *m/z*: $[M-H]^-$ calculated for $C_{10}H_{19}O_3P$, 217.0999, found 217.0984.

**((4-Isopropylcyclohex-1-en-1-yl)methyl)phosphonic acid 12**[3]

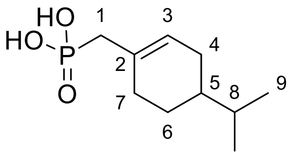


β-Pinene **10** (1 eq., 20 mmol, 3.1 mL) was dissolved in 140 mL of dry methanol. $NH_4H_2PO_2$ (2.5 eq., 50 mmol, 4.2 g) and triethylborane (1 M in THF, 2.1 eq., 21 mL) were added. The reaction mixture was vigorously stirred under air for 4 h. All volatile components were removed under reduced pressure and the residue was dissolved in 50 mL EtOAc and 50 mL 2 M aqueous HCl. The aqueous layer was extracted two times with 50 mL EtOAc. The combined organic layers were dried over $Na_2SO_4$, filtered and the solvent removed under reduced pressure. The residue was dissolved in 50 mL THF. DMSO (1.1 eq., 23 mmol, 1.6 mL) and iodine (0.01 eq., 0.2 mmol, 48 mg) were added. The reaction mixture was heated to 60 °C for 3 h. ($H_2O$:MeCN 95:5 V./V. → 5:95 V./V., 0.1% formic acid). Freeze drying gave 2.92 g (13.4 mmol, 60%) of the title compound **12** as colorless solid.

$^1$H NMR (400 MHz, MeOD) $\delta$ = 5.58 (s, 1 H, 3-H), 2.45 (d, $^1J$ = 21.7 Hz, 2 H, 1-H), 2.16 (s, 2 H, 6-H), 2.12 - 2.02 (m, 1 H, 4-Ha), 1.84 – 1.72 (m, 2 H, 4-Hb, 7-Ha), 1.47 (m, 1 H, 8-H), 1.33 – 1.20 (m, 2 H, 5-H, 7-Hb), 0.91 (t, $^3J$ = 6.4 Hz, 6 H, 9-H).

$^{13}C$ NMR (100 MHz, MeOD) $\delta$ = 130.4 (d, $^{2}J$ = 10.9 Hz, C2), 127.6 (d, $^{3}J$ = 12.4 Hz, C3), 41.1 (C5), 37.2 ($^{1}J$ = 135.1 Hz, C1), 33.4 (C8), 31.2 (d, $^{4}J$ = 2.8 Hz, C6), 30.3 (d, $^{4}J$ = 2.7 Hz, C4), 27.6 (C7), 20.3 (C9a), 20.1 (C9b).

$^{31}P$ NMR (162 MHz MeOD) $\delta$ = 27.33 (s).

HRMS-ESI *m/z*: $[M-H]^-$ calculated for $C_{10}H_{19}O_3P$, 217.0999, found 217.0985

**(2-Methyl-5-(1-phosphonopropan-2-yl)cyclohexyl)phosphonic acid 14**

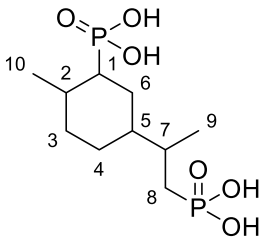


Limonene **13** (1 eq., 61.7 mmol, 10.0 mL.) was dissolved in diethylphosphite (5.75 eq., 355 mmol, 45.7 mL) under argon atmosphere. The reaction mixture was degassed under argon for 15 minutes. Di-*tert*-butyl peroxide (0.06 eq., 4 mmol, 700 µL) was added. The reaction mixture was heated to 125 °C for 24 h. Another 0.06 eq Di-*tert*-butyl peroxide were added after 2 h, 16 h and 20 h. The reaction mixture was cooled to room temperature and the volatile components were removed in vacuo. The residue was dissolved in 300 mL $Et_2O$ and washed two times with 200 mL saturated $NaHCO_3$ solution. Filtration and removal of the solvent gave 22.7 g (55.0 mmol, 89%) of intermediate diethyl (5-(1-(diethoxyphosphoryl)propan-2-yl)-2-methylcyclohexyl)phosphonate[4] as a yellow oil.

$^{1}H$ NMR (400 MHz, $CDCl_3$) $\delta$ = 4.17 – 3.98 (m, 8 H, 11-H, 13-H), 2.18 – 1.35 (m, 10 H), 1.30 (t, $^{3}J$ = 7.0 Hz, 12 H, 12-H, 14-H), 1.26 – 0.95 (m, 8 H).

$^{31}P$ NMR (162 MHz $CDCl_3$) $\delta$ = 33.63 (d), 32.94 (s), 32.87 (s), 32.78 (s), 32.59 (s), 32.52 (s), 32.44 (s), 32.04 (d).HRMS-ESI *m/z*: $[M+H]^+$ calculated for $C_{18}H_{38}O_6P_2$, 413.2216, found 413.2237.

This intermediate ester (1 eq., 33.3 mmol, 10.0 g) was dissolved in 240 mL aqueous HCl (6 M, 1.44 mol). The reaction mixture was heated to 100 °C for 48 h. The solvent was removed under reduced pressure. Freeze drying gave 7.69 g (25.6 mmol, 77%) of the title compound **14** as a colorless, hygroscopic solid.

$^{31}P$ NMR (162 MHz MeOD) $\delta$ = 32.72 (s), 32.42 (s), 31.74 (s), 31.64 (s), 31.07 (s), 30.94 (d), 30.64 (s), 30.31 (s).

HRMS-ESI *m/z*: $[M-H]^-$ calculated for $C_{10}H_{22}O_6P_2$, 299.0819, found 299.0818.

**((4-Isopropyl-2-phosphonocyclohexyl)methyl)phosphonic acid 15**

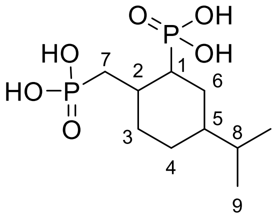


Pinene **10** ( 1 eq., 96.0 mmol, 15.0 mL) was dissolved in diethylphosphite (3 eq., 288 mmol, 37.2 mL) under argon atmosphere. The reaction mixture was degassed under argon for 15 minutes. Di-*tert*-butyl peroxide (0.1 eq., 9.6 µmol, 1.8 mL) was added. The reaction mixture was heated to 125 °C for 18 h. The reaction mixture was cooled to room temperature and the volatile components were removed under oil pump vacuum. The residue was dissolved in 700 mL $Et_2O$ and washed two times with 40 mL saturated $NaHCO_3$ solution. Filtration and removal of the solvent gave 29.0 g (70.3 mmol, 73%) of intermediate diethyl ((2-(diethoxyphosphoryl)-4-isopropylcyclohexyl)methyl)phosphonate as a yellow oil.

$^1H$ NMR (400 MHz, $CDCl_3$) $\delta$ = 5.17 – 3.97 (m, 8 H, 10-H, 12-H), 2.51 – 1.33 (m, 10 H), 1.33 – 1.25 (m, 12 $^{31}P$ NMR (162 MHz $CDCl_3$) $\delta$ = 32.67 (s), 32.39 (d), 32.13 (d), 31.84 (d), 31.73 (d), 31.45 (d), 31.23 (s), 31.98 (d).

HRMS-ESI *m/z*: $[M+H]^+$ calculated for $C_{18}H_{38}O_6P_2$, 413.2216, found 413.2194.

This intermediate ester (1 eq., 12.1 mmol, 5.00 g) was dissolved in 100 mL aqueous HCl (6 M, 600 mmol). The reaction mixture was heated to 100 °C for 40 h. The solvent was removed under reduced pressure. Freeze drying gave 3.56 g (11.9 mmol, 98%) of the title compound **15** as a colorless, hygroscopic solid.

$^{31}P$ NMR (162 MHz MeOD) $\delta$ = 31.17 (d), 30.60 (d), 30.47 (d), 30.22 (d), 29.90 (d), 29.77 (d), 29.67 (d), 29.39 (d).

HRMS-ESI *m/z*: $[M-H]^-$ calculated for $C_{10}H_{22}O_6P_2$, 299.0819, found 299.0787.

**(4,4a-Dimethyl-6-(1-phosphonopropan-2-yl)decahydronaphthalen-1-yl)phosphonic acid 16**

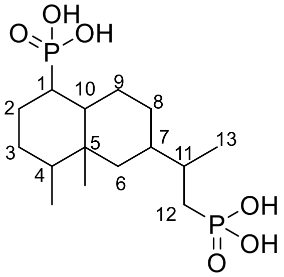


Valencene (1 eq., 14.6 mmol, 2.99 mL) was dissolved in diethylphosphite (6 eq., 87.8 mmol, 11.3 mL) under argon atmosphere. The reaction mixture was degassed under argon for 15 minutes. Di-*tert*-butyl peroxide (0.1 eq., 1.5 µmol, 270 µL) was added. The reaction mixture was heated to 125 °C for 24 h. Another 0.1 eq Di-*tert*-butyl peroxide were added after 3 h, 17 h and 21 h. The reaction mixture was cooled to room temperature and the volatile components were removed under oil pump vacuum. The residue was dissolved in 250 mL $Et_2O$ and washed two times with 200 mL saturated $NaHCO_3$ solution. Filtration and removal of the solvent gave 6.70 g (13.9 mmol, 95%) of intermediate diethyl (6-(1-(diethoxyphosphoryl)propan-2-yl)-4,4a-dimethyldecahydronaphthalen-1-yl)phosphonate as a yellow oil.

$^1$H NMR (400 MHz, MeOD) $\delta$ = 4.15 – 4.01 (m, 8 H, 16-H), 2.18 – 1.41 (m, 13 H), 1.36 – 1.28 (m, 12 $^{31}$P NMR (162 MHz $CDCl_3$) $\delta$ = 34.98 (s), 34.41 (s), 34.30 (s).

HRMS-ESI *m/z*: $[M+H]^+$ calculated for $C_{23}H_{46}O_6P_2$, 481.2842, found 481.2876.

This intermediate ester (1 eq., 10.4 mmol, 5.00 g) was dissolved in 120 mL aqueous HCl (6 M, 720 mmol). The reaction mixture was heated to 100 °C for 48 h. The solvent was removed under reduced pressure. The residue was dissolved in 150 mL of 2 M aqueous NaOH and washed with 150 mL $Et_2O$ three times. The pH value was adjusted to 1 with aqueous HCl and extracted with 150 mL of EtOAc three times. The combined organic layers were dried over $Na_2SO_4$, filtered and the solvent removed under reduced pressure. Freeze drying gave 2.18 g (5.92 mmol, 57%) of the title compound **16** as a colorless solid.

$^{31}$P NMR (162 MHz $CDCl_3$) $\delta$ = 32.16 (s), 32.07 (s), 31.04 (s), 30.99 (s), 30.94 (s), 30.87 (s), 29.57 (s).

HRMS-ESI *m/z*: $[M-H]^-$ calculated for $C_{15}H_{30}O_6P_2$, 367.1445, found 367.1444

**10-Phosphonooctadecanoic acid 18**[5]

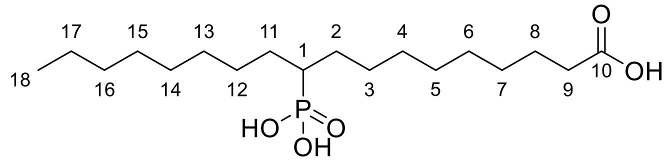


To oleic acid **17** (90%, 1 eq., 159 mmol, 50.0 g) diethylphosphite (3 eq. 478 mmol, 61.7 mL) was added under argon atmosphere. The solution was degassed for 30 minutes. Di-*tert*-butyl peroxide (0.1 eq., 16.0 mmol, 2.93 mL) was added. The reaction mixture was heated to 125 °C for 24 h. Another 0.1 eq Di-*tert*-butyl peroxide were added after 2 h, 16 h and 20 h. The reaction mixture was cooled to room temperature and the volatile components were removed under oil pump vacuum. The residue was dissolved in 500 mL EtOAc and washed twice with 500 mL saturated $Na_2CO_3$ solution. The organic layer was dried over $Na_2SO_4$ and filtered. Removal of the solvent under reduced pressure gave 65.2 g (155 mmol, 97%) of intermediate 10-(diethoxyphosphoryl)octadecanoic acid as a pale yellow oil.

$^1$H NMR (300 MHz, $CDCl_3$) $\delta$ = 4.15 – 4.02 (m, 4 H, 20-H), 2.27 (t, $^3J$ = 7.6 Hz, 2 H, 9-H), 1.75 – 1.53 (m, 5 H, $CH_2$), 1.53 – 1.18 (m, 31 H, $CH_2$, 21-H), 0.87 (t, $^3J$ = 6.7 Hz, 3 H, 18-H).

$^{13}$C NMR (100 MHz, $CDCl_3$) $\delta$ = 174.0 (d, $^{10}J$ = 3.3 Hz, C10), 61.5 (C20), 60.3 ($CH_2$), 36.1 (d, $^1J$ = 137 Hz, C1), 34.5 ($CH_2$), 34.1 ($CH_2$), 32.0 ($CH_2$), 29.9 ($CH_2$), 29.7 ($CH_2$), 29.6 ($CH_2$), 29.5 ($CH_2$), 29.4 ($CH_2$), 29.3 ($CH_2$), 29,1 ($CH_2$), 27.8 ($CH_2$), 27.7 ($CH_2$), 25.1 ($CH_2$), 24.9 ($CH_2$), 22.8 ($CH_2$), 16.6 (d, $^3J$ = 5.8 Hz, C21), 14.3 (d, $^9J$ = 22.5 Hz, C18).

$^{31}$P NMR (162 MHz $CDCl_3$) $\delta$] = 35.11 (s), 35.06 (s).

HRMS-ESI *m/z*: $[M+H]^+$ calculated for $C_{22}H_{45}O_5P$, 421.3077, found 421.3092.

This intermediate ester (1 eq., 143 mmol, 60.0 g) was dissolved in a mixture of 300 mL dioxane and 300 mL aqueous HCl (12 m, 3.2 mol). The reaction mixture was heated to 100 °C for 24 h. The organic solvent was removed under reduced pressure. The residue was dilluted with 600 mL water. The pH value was adjusted to 12 with solid NaOH. The aqueous layer was washed with 600 mL EtOAc three times. The pH value was adjusted to 1 with concentrated aqueous HCl. The aqueous layer was extracted three times with 600 mL $Et_2O$. The combined

organic layers were dried over $Na_2SO_4$ and filtered. Removal of the solvent under reduced pressure and coevaporating five times with 50 mL $Et_2O$ gave 47.6 g (130 mmol, 91%) of the title compound **18** as amorphous, waxy solid.

$^1$H NMR (300 MHz, $CDCl_3$) $\delta$ = 7.61 ($s_{br}$, 2 H, POOH), 2.34 (t, $^3J$ = 7.1 Hz, 2 H, 9-H), 1.80 – 1.57 (m, 5 H, $CH_2$), 1.57 – 1.21 (m, 24 H, $CH_2$), 0.88 (t, $^3J$ = 6.8 Hz, 3 H, 18-H).

$^{13}$C NMR (100 MHz, $CDCl_3$) $\delta$ = 180.1 (C10), 35.6 (d, $^1J$ = 140 Hz, C1), 34.2 ($CH_2$), 32.1 ($CH_2$), 29.9 ($CH_2$), 29.7 ($CH_2$), 29.6 ($CH_2$), 29.5 ($CH_2$), 29.1 ($CH_2$), 28.9 ($CH_2$), 28.1 ($CH_2$), 28.0 ($CH_2$), 27.9 ($CH_2$), 27.7 ($CH_2$), 27.6 ($CH_2$), 24.7 ($CH_2$), 22.8 ($CH_2$), 14.3 (C18).

$^{31}$P NMR (162 MHz $CDCl_3$) $\delta$ = 40.45 (s), 40.32 (s).

HRMS-ESI *m/z*: $[M-H]^-$ calculated for $C_{18}H_{37}O_5P$, 363.2306, found 363.2296.

**10-(Diethoxyphosphoryl)octadec-9-enoic acid 23**

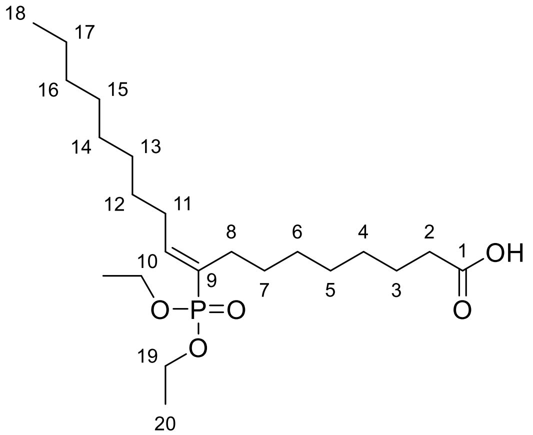


To ricinolic acid **19** (80%, 1 eq., 10.1 mmol, 400 g) diethylphosphite (5 eq., 50.4 mmol, 6.50 mL) was added under argon atmosphere. The solution was degassed for 15 minutes. Di-*tert*-butyl peroxide (0.1 eq., 1.0 mmol, 190 µL) was added. The reaction mixture was heated to 125 °C for 24 h. Another 0.1 eq Di-*tert*-butyl peroxide were added after 2 h and 17 h. The reaction mixture was cooled to room temperature and the volatile components were removed under oil pump vacuum. The residue was dissolved in 150 mL $Et_2O$ and washed twice with 100 mL saturated $Na_2CO_3$ solution. The organic layer was dried over $Na_2SO_4$ and filtered. Removal of the solvent under reduced pressure gave 3.95 g (9.40 mmol, 94%) of the ester **23** as a pale yellow oil.

$^1$H NMR (400 MHz, $CDCl_3$) $\delta$ = 4.32 – 3.95 (m, 5 H, 10-H, 19-H), 2.19 (t, $^3J$ = 7.5 Hz, 2 H, 2-H), 2.06 – 1.12 (m, 34 H, 20-H, $CH_2$), 0.80 (t, $^3J$ = 6.9 Hz, 3 H, 18-H).

$^{31}$P NMR (162 MHz, $CDCl_3$) $\delta$ = 50.59 (s), 48.91 (s), 48.03 (s), 46.98 (s), 29.91 (s), 28.89 (s), 28.06 (s), 27.50 (s).

HRMS-ESI *m/z*: $[M+H]^+$ calculated for $C_{22}H_{43}O_5P$, 419.2921, found 419.2936.

**10-Phosphonooctadec-9-enoic acid 24**

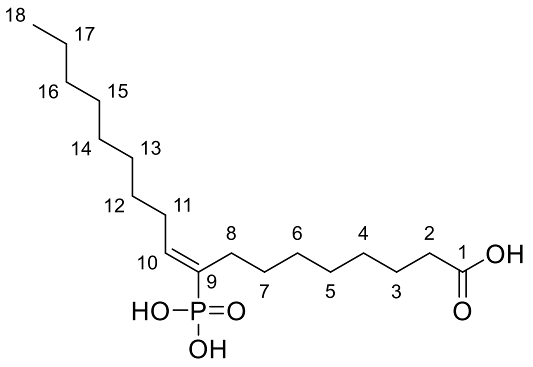


Ester **23** (1 eq., 2.39 mmol, 1.00 g) was dissolved in a mixture of 75 mL dioxane and 75 mL aqueous HCl (12 m, 0.9 mol). The reaction mixture was heated to 100 °C for 48 h. The organic solvent was removed under reduced pressure. The residue was dissolved in 100 mL 2 N NaOH. The aqueous layer was washed with 100 mL EtOAc three times. The pH value was adjusted to 1 with concentrated aqueous HCl. The aqueous layer was extracted three times with 100 mL $Et_2O$. The combined organic layers were dried over $Na_2SO_4$ and filtered. Removal of the solvent under reduced pressure and repeated coevaporation with 20 mL $Et_2O$ (5 times) gave 650 mg (1.79 mmol, 75%) of the title compound **24** as yellow waxy solid.

$^1$H NMR (400 MHz, $CDCl_3$) $\delta$ = 9.42 ($s_{br}$ 3 H, -OH), 4.42 – 3.76 (m, 1 H, 10-H), 2.20 – 1.18 (m, 26 H, $CH_2$), 0.88 (t, $^3J$ = 6.0 Hz, 3 H, 18 H).

$^{31}$P NMR (162 MHz, $CDCl_3$) $\delta$ = 52.08 ($s_{br}$), 50.78 ($s_{br}$), 32.41 ($s_{br}$), 32.19 ($s_{br}$).

HRMS-ESI *m/z*: $[M-H]^-$ calculated for $C_{18}H_{35}O_5P$, 361.2149, found 361.2082.

**Diethyl (3,7-dimethyloct-6-en-1-yl)phosphonate 26**

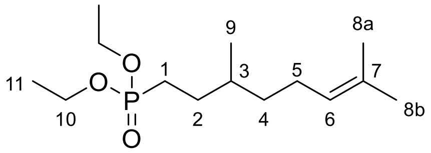


Citronellol **25** (1 eq., 2.0 mL, 11 mmol) and $CBr_4$ (1.1 eq., 4.0 g, 12 mmol) were dissolved in 2 mL $CH_2Cl_2$. Triphenylphosphine (1.2 eq, 3.5 g, 13 mmol) dissolved in 2 mL $CH_2Cl_2$ was added at 0 °C. The reaction mixture was stirred at room temperature for 5 h. The solvent was removed under reduced pressure. Purification by chromatography on silica (pentane) gave 2.03 g (9.35 mmol, 85%) 8-bromo-2,6-dimethyloct-2-ene[6] as a colorless oil.

$^{1}$H NMR (300 MHz, $CDCl_3$) $\delta$ = 5.09 (t, $^{3}J$ = 7.1 Hz, 1 H, 6-H), 3.5 – 3.37 (m, 2 H, 1-H), 2.08 - 1.80 (m, 3 H, 2-H, 5a-H), 1.74 – 1.52 (m, 8 H, 9a-H, 9b-H, 3-H, 5b-H), 1.32 – 1.08 (m, 2 H, 4-H), 0.9 (d, $^{3}J$ = 6.3 Hz, 3 H, 9-H).

$^{13}$C NMR (150 MHz, $CDCl_3$) $\delta$ = 131.6 (C7), 124.6 (C6), 40.2 (C5), 36.8 (C4), 32.3 (C1), 31.5 (C3), 25.9 (C8b), 25.5 (C2), 19.0 (C9), 17.8 (C8a).

This intermediate bromide (1 eq., 2.0 g, 9.3 mmol) was dissolved in triethyl phosphite (6.2 eq, 10 mL, 58 mmol). The reaction mixture was stirred at 150 °C for 12 h. The solvent was removed under reduced pressure. Purification by chromatography on silica (pentane:EtOAc 1:1 V/V → EtOAc) gave 1.06 g (3.86 mmol, 41%) of the title compound **26** as a colorless oil.

$^{1}$H NMR (300 MHz, $CDCl_3$) $\delta$ = 5.07 (t, $^{3}J$ = 7.1 Hz, 1 H, 6-H), 4.18 – 4.00 (m, 4 H, 10-H), 2.05 – 1.90 (m, 2 H, 5-H), 1.89 – 1.51 (m, 8 H, 8-H, 1-H), 1.51 – 1.37 (m, 2 H, 2-H), 1.36 – 1.07 (m, 2 H, 4-H), 1.32 (t, $^{3}J$ = 7.1 Hz, 7 H, 11-H, 3-H), 0.88 (d, $^{3}J$ = 6.5 Hz, 3 H, 9-H).

$^{13}$C NMR (150 MHz, $CDCl_3$) $\delta$ = 131.4 (C7), 124.6 (C6), 61.5 (C10), 36.4 (C4), 33.1 (C3), 29.4 (d, $^{1}J$ = 140 Hz, C1), 29.1 (d, $^{2}J$ = 5.3 Hz, C2), 25.7 (C8b), 25.4 (C5), 18.9 (C9), 17.6 (C8a), 16.5 (d, $^{3}J$ = 4.8 Hz, C11).

$^{31}$P NMR (162 MHz $CDCl_3$) $\delta$ = 33.31 (s).

**(3,7-Dimethyloct-6-en-1-yl)phosphonic acid 27**

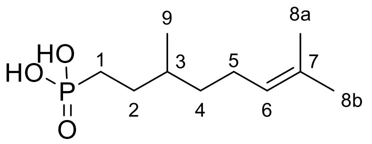


Phosphonate **26** (1 eq., 400 mg, 1.45 mmol) was dissolved in 5 mL absolute $CH_2Cl_2$ under argon atmosphere. Bromotrimethylsilane (4 eq., 770 µL, 5.8 mmol) was added dropwise. The reaction mixture was stirred for 30 min at room temperature. All volatile components were removed under reduced pressure. 20 mL of a mixture of water and THF (1:1 V/V) was added. The reaction mixture was stirred for 2 h at room temperature. The organic solvent was removed under reduced pressure. After freeze drying, purification (H$_2$O:MeCN 98:2 V/V, 0.1% formic acid → H$_2$O:MeCN 2:98 V/V, 0.1% HCOOH) and consecutive freeze drying gave 296 mg (1.34 mmol, 92%) of the title compound **27** as a colorless solid.

$^1$H NMR (600 MHz, MeOD) $\delta$ = 5.11 (t, $^3J$ = 7.1 Hz, 1 H, 6-H), 2.08 – 1.93 (m, 2 H, 5-H), 2.15 – 1.57 (m, 9 H, 8a-H, 8b-H, 1-H, 2a-H), 1.52 – 1.39 (m, 2 H, 2b-H, 3-H), 1.39 – 1.32 (m, 1 H, 4a-H), 1.22 – 1.14 (m, 1 H, 4b-H), 0.92 (d, $^3J$ = 6.5 Hz, 3 H, 9-H).

$^{13}$C NMR (150 MHz, MeOD) $\delta$ = 132.1 (C7), 125.7 (C6), 37.7 (C4), 34.34 (d, $^3J$ = 16.8 Hz, C3), 30.7 (d, $^2J$ = 4.8 Hz, C2), 26.4 (C5), 25.9 (C8b), 25.8 (d, $^1J$ = 138 Hz, C1), 19.5 (C9), 17.71 (C8a).

$^{31}$P NMR (162 MHz, MeOD) $\delta$ = 30.67 (s).

HRMS-ESI *m/z*: [M-H]$^-$ calculated for $C_{10}H_{20}O_3P$, 219.1156, found 219.1144

**(3,7-Dimethyloctyl)phosphonic acid 28**

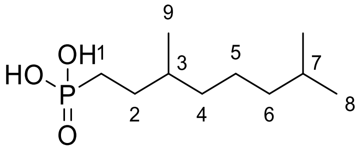


Unsaturated phosphonate **26** (1 eq., 400 mg, 1.45 mmol) was dissolved in 5 mL methanol and Pd/C (10%, 0.1 eq, 555 mg, 145 µmol) was added. The reaction mixture was stirred under $H_2$ atmosphere for 16 h and filtered over Celite®. Removal of the solvent under reduced pressure gave 406 mg (1.45 mmol, quant.) diethyl (3,7-dimethyloctyl)phosphonate as an intermediate (colorless oil), which was used in the next step without further purification.

$^{1}H$ NMR (600 MHz, $CDCl_3$) $\delta$ = 4.14 – 4.03 (m, 4 H, 11-H), 1.80 – 1.55, (m, 3 H, 1-H, 2-Ha), 1.51 (hep, $^{3}J$ = 6.7 Hz, 1 H, 7-H), 1.47 – 1.36 (m, 2 H, 2-Hb, 7 H), 1.32 (t, $^{3}J$ = 7.1 Hz, 6 H, 10-H), 1.30 – 1.19 (m, 3 H, 5-H, 4-Ha), 1.16 – 1.05 (m, 3 H, 4-Hb, 6-H), 0.88 – 0.83 (m, 9 H, 8-H, 9-H).

$^{13}C$ NMR (150 MHz, $CDCl_3$) $\delta$ = 61.6 (C10), 39.4 (C6), 37.7 (C4), 33.6 (d, $^{3}J$ = 16.8 Hz, C3), 29.3 (d, $^{2}J$ = 5 Hz, C2), 28.8 (C7), 24.8 (C5), 23.5 (d, $^{1}J$ = 140 Hz, C1), 22.9 (d, $^{4}J$ = 14.3 Hz, C9), 19.2 (C8), 16.6 (d, $^{3}J$ = 6 Hz, C11).

$^{31}P$ NMR (162 MHz $CDCl_3$) $\delta$ = 33.33 (s).

This intermediate diethylester (1 eq., 406 mg, 1.45 mmol) was dissolved in 5 mL absolute $CH_2Cl_2$ under argon atmosphere. Bromotrimethylsilane (4 eq., 470 µL, 5.8 mmol) was added dropwise. The reaction mixture was stirred for 30 min at room temperature. All volatile components were removed under reduced pressure. 20 mL of a mixture of water and THF (1:1 V/V) was added. The reaction mixture was stirred for 2 h at room temperature. The organic solvent was removed under reduced pressure. Freeze drying gave 301 mg (1.36 mmol, 94%) of the title compound **28** as a colorless solid.

$^{1}H$ NMR (600 MHz, MeOD) $\delta$ = 1.75 – 1.59 (m, 3 H, 1-H, 2-Ha), 1.54 (hep, $^{3}J$ = 6.7 Hz, 1 H, 7-H), 1.45 – 1.27 (m, 5 H, 2-Hb, 3-H, 5-Ha, 4-H), 1.21 – 1.10 (m, 3 H, 5-Hb, 6-H), 0.92 – 0.87 (m, 9 H, 8-H, 9-H).

$^{13}C$ NMR (150 MHz, MeOD) $\delta$ = 40.5 (C6), 37.9 (C4), 34.7 (d, $^{3}J$ = 16 Hz, C3), 30.8 (d, $^{2}J$ = 5 Hz, C2), 29.2 (C7), 25.8 (C5), 25.7 (d, $^{1}J$ = 139 Hz, C1), 23.0 (d, $^{4}J$ = 13 Hz, C9), 19.5 (C8).

$^{31}P$ NMR (162 MHz, MeOD) $\delta$ = 30.69 (s).

HRMS-ESI *m/z*: $[M-H]^-$ calculated for $C_{10}H_{23}O_3P$, 221.1312, found 221.1337

# CMC Evaluations

### Experimental procedure

350 µmol of the acid and 1.5 equivalents of the basic additive per acidic proton of the acid were dissolved in 10 mL of $D_2O$ in a volumetric flask. If necessary, the pH value was adjusted to 8.5 with NaOD. An NMR sample was prepared. 6 mL of the solution were added to a second volumetric flask and diluted to 10 mL with $D_2O$. The process was repeated until 10 samples with the concentrations of 35 mmol/L, 21 mmol/L, 13 mmol/L, 7.6 mmol/L, 4.5 mmol/L, 2.7 mmol/L, 1.6 mmol/L, 1.0 mmol/L, 0.59 mmol/L and 0.35 mmol/L were obtained. A suitable peak of the NMR spectrum that showed a noticeable concentration dependent shift was identified and its shift plotted against the inverse concentration. The inflection point indicating the CMC was determined *via* graphical methods.

### Measured CMCs (Table S1)

**Table S1:** CMC values determined *via* $^{1}$H-NMR.

| Substance | No Additive | TEA |
|---|---|---|
| **OPA** | 2.8 mmol/L | 4.7 mmol/L |
| **TC®** | 4.8 mmol/L | 8.9 mmol/L |
| **3** | 4.5 mmol/L | 4.7 mmol/L |
| **12** | 7.5 mmol/L | 4.7 mmol/L |
| **18** | 30 mmol/L | 19.1 mmol/L |

# NMR Spectra

## OPA

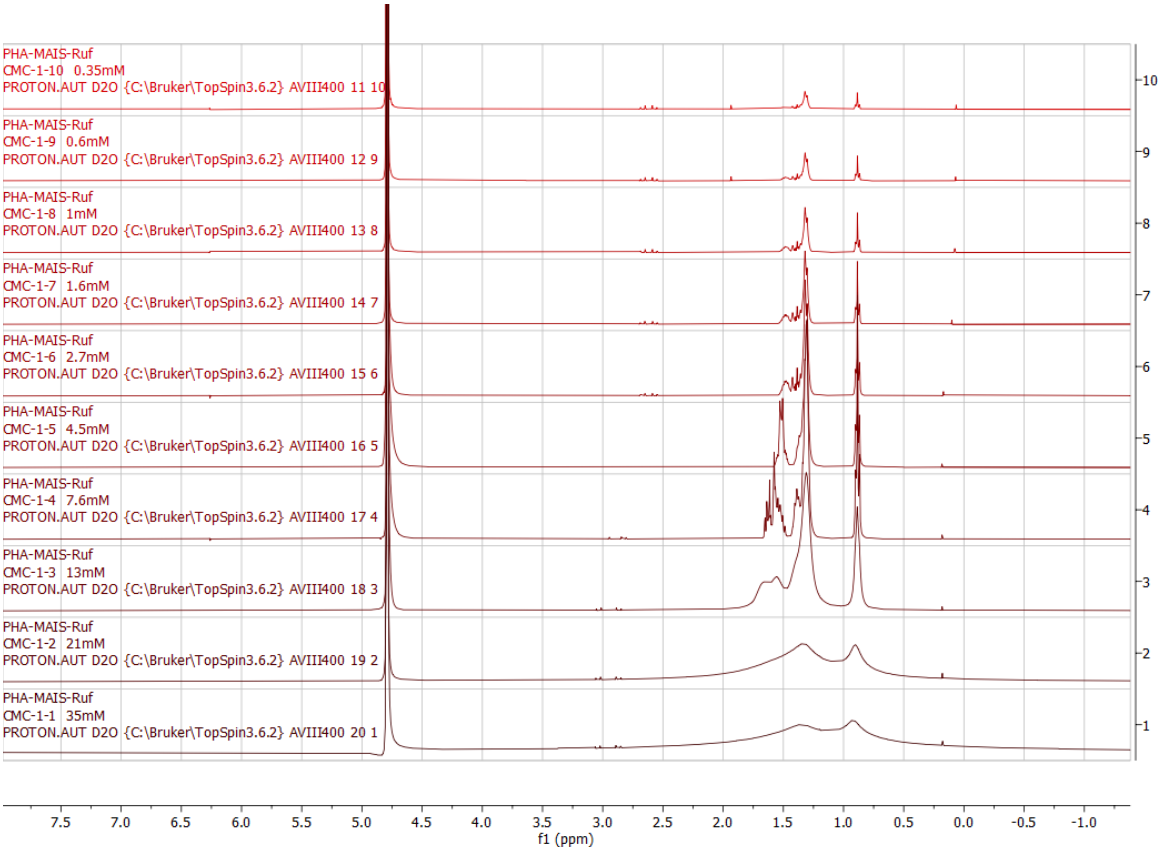


## OPA + TEA

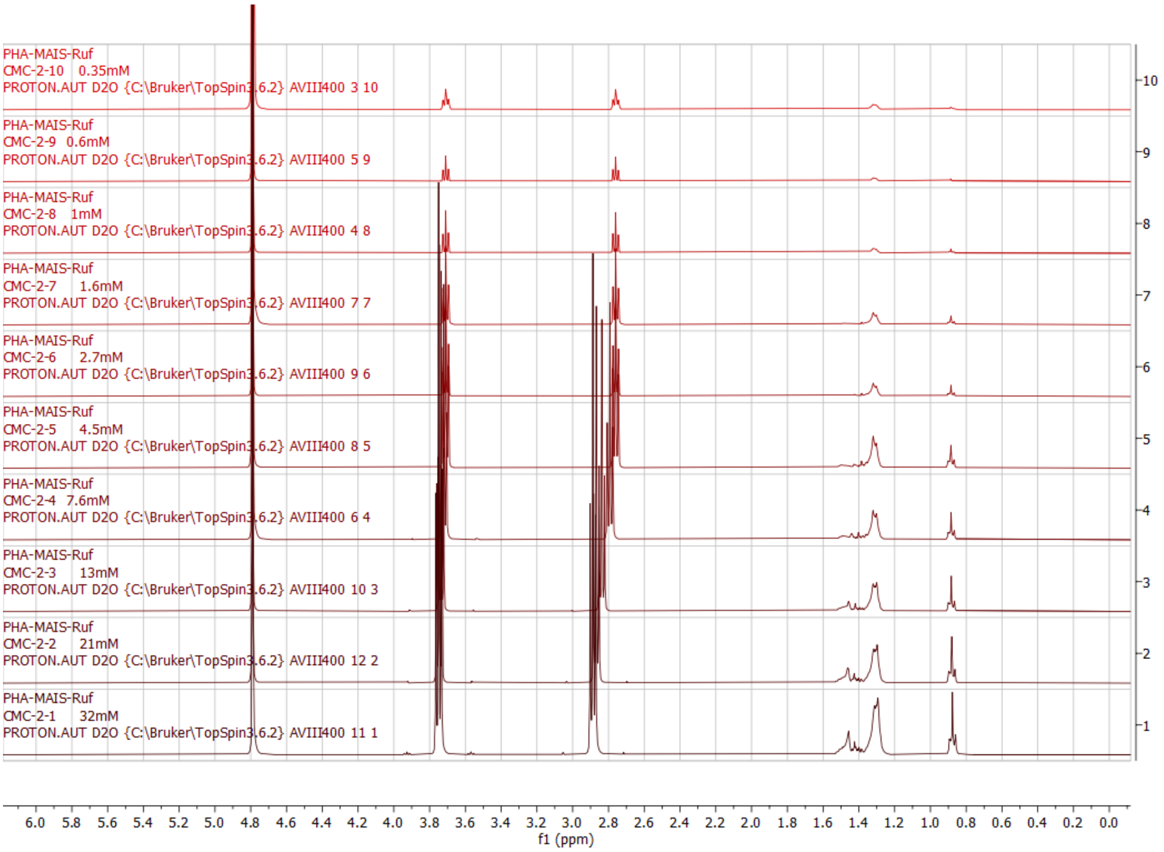

**TC®**

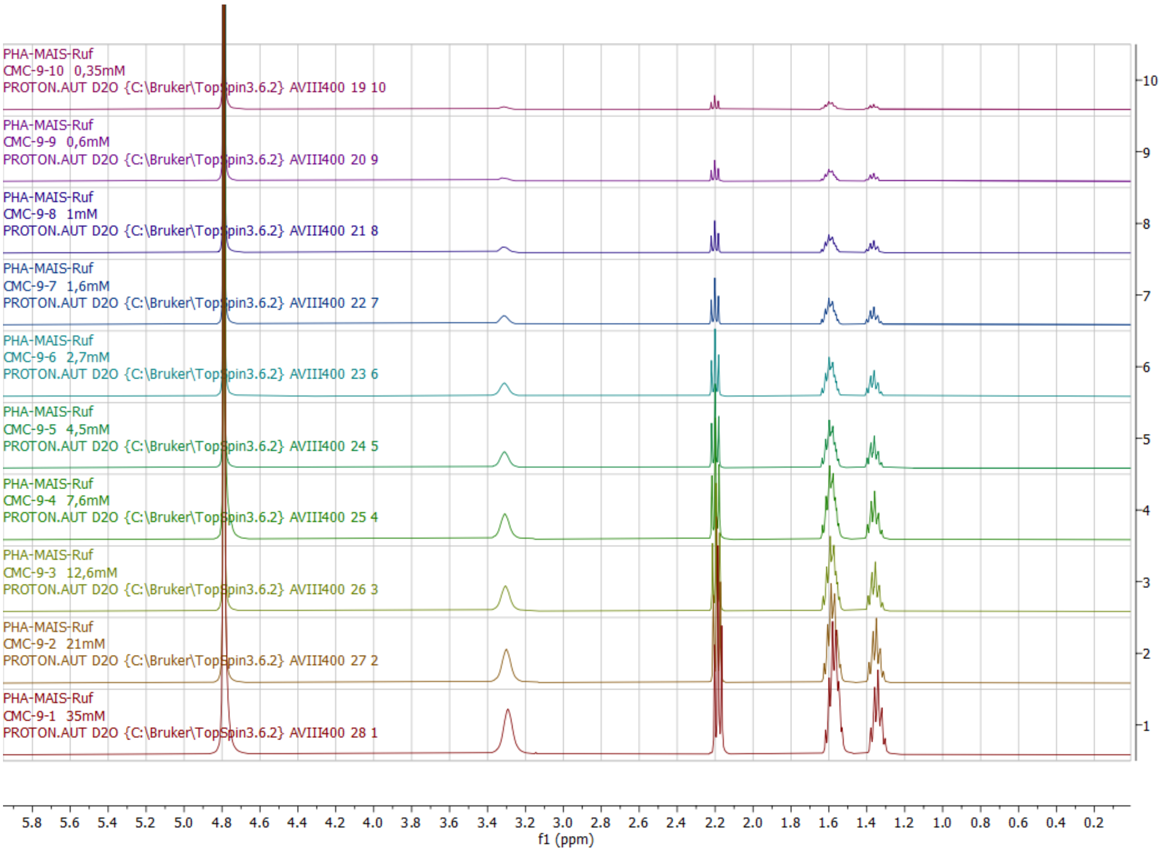

PHA-MAIS-Ruf
CMC-9-10 0,35mM
PROTON.AUT D2O {C:\Bruker\TopSpin3.6.2} AVIII400 19 10
PHA-MAIS-Ruf
CMC-9-9 0,6mM
PROTON.AUT D2O {C:\Bruker\TopSpin3.6.2} AVIII400 20 9
PHA-MAIS-Ruf
CMC-9-8 1mM
PROTON.AUT D2O {C:\Bruker\TopSpin3.6.2} AVIII400 21 8
PHA-MAIS-Ruf
CMC-9-7 1,6mM
PROTON.AUT D2O {C:\Bruker\TopSpin3.6.2} AVIII400 22 7
PHA-MAIS-Ruf
CMC-9-6 2,7mM
PROTON.AUT D2O {C:\Bruker\TopSpin3.6.2} AVIII400 23 6
PHA-MAIS-Ruf
CMC-9-5 4,5mM
PROTON.AUT D2O {C:\Bruker\TopSpin3.6.2} AVIII400 24 5
PHA-MAIS-Ruf
CMC-9-4 7,6mM
PROTON.AUT D2O {C:\Bruker\TopSpin3.6.2} AVIII400 25 4
PHA-MAIS-Ruf
CMC-9-3 12,6mM
PROTON.AUT D2O {C:\Bruker\TopSpin3.6.2} AVIII400 26 3
PHA-MAIS-Ruf
CMC-9-2 21mM
PROTON.AUT D2O {C:\Bruker\TopSpin3.6.2} AVIII400 27 2
PHA-MAIS-Ruf
CMC-9-1 35mM
PROTON.AUT D2O {C:\Bruker\TopSpin3.6.2} AVIII400 28 1
f1 (ppm)


**TC®** + TEA

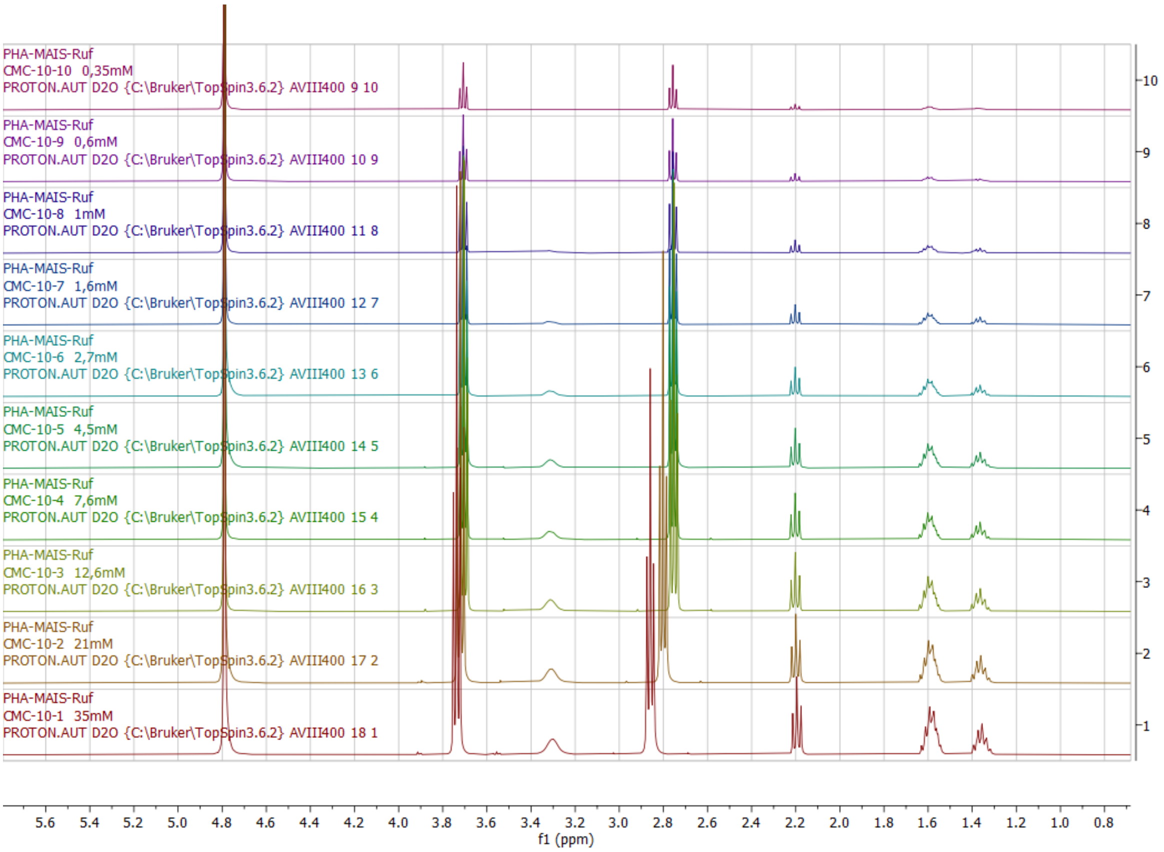

PHA-MAIS-Ruf
CMC-10-10 0,35mM
PROTON.AUT D2O {C:\Bruker\TopSpin3.6.2} AVIII400 9 10
PHA-MAIS-Ruf
CMC-10-9 0,6mM
PROTON.AUT D2O {C:\Bruker\TopSpin3.6.2} AVIII400 10 9
PHA-MAIS-Ruf
CMC-10-8 1mM
PROTON.AUT D2O {C:\Bruker\TopSpin3.6.2} AVIII400 11 8
PHA-MAIS-Ruf
CMC-10-7 1,6mM
PROTON.AUT D2O {C:\Bruker\TopSpin3.6.2} AVIII400 12 7
PHA-MAIS-Ruf
CMC-10-6 2,7mM
PROTON.AUT D2O {C:\Bruker\TopSpin3.6.2} AVIII400 13 6
PHA-MAIS-Ruf
CMC-10-5 4,5mM
PROTON.AUT D2O {C:\Bruker\TopSpin3.6.2} AVIII400 14 5
PHA-MAIS-Ruf
CMC-10-4 7,6mM
PROTON.AUT D2O {C:\Bruker\TopSpin3.6.2} AVIII400 15 4
PHA-MAIS-Ruf
CMC-10-3 12,6mM
PROTON.AUT D2O {C:\Bruker\TopSpin3.6.2} AVIII400 16 3
PHA-MAIS-Ruf
CMC-10-2 21mM
PROTON.AUT D2O {C:\Bruker\TopSpin3.6.2} AVIII400 17 2
PHA-MAIS-Ruf
CMC-10-1 35mM
PROTON.AUT D2O {C:\Bruker\TopSpin3.6.2} AVIII400 18 1
f1 (ppm)

**3**

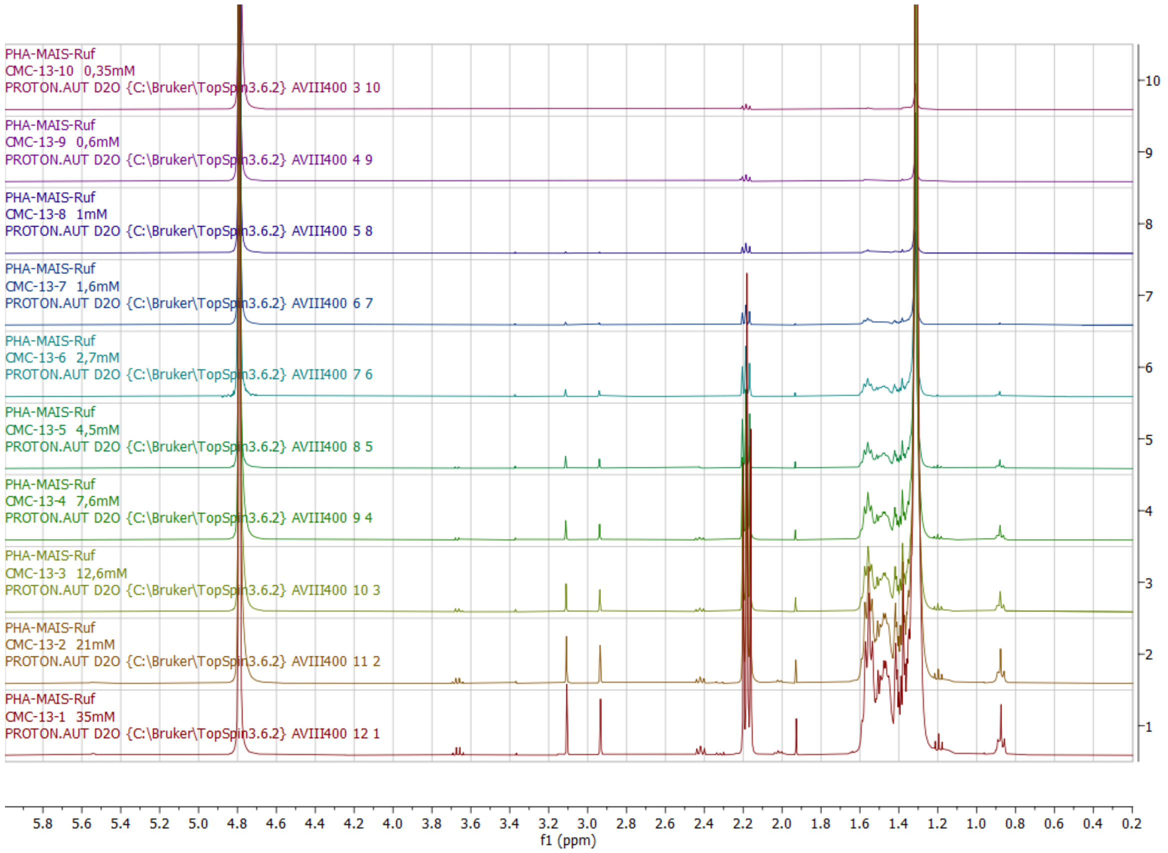
PHA-MAIS-Ruf
CMC-13-10 0,35mM
PROTON.AUT D2O {C:\Bruker\TopSpin3.6.2} AVIII400 3 10
PHA-MAIS-Ruf
CMC-13-9 0,6mM
PROTON.AUT D2O {C:\Bruker\TopSpin3.6.2} AVIII400 4 9
PHA-MAIS-Ruf
CMC-13-8 1mM
PROTON.AUT D2O {C:\Bruker\TopSpin3.6.2} AVIII400 5 8
PHA-MAIS-Ruf
CMC-13-7 1,6mM
PROTON.AUT D2O {C:\Bruker\TopSpin3.6.2} AVIII400 6 7
PHA-MAIS-Ruf
CMC-13-6 2,7mM
PROTON.AUT D2O {C:\Bruker\TopSpin3.6.2} AVIII400 7 6
PHA-MAIS-Ruf
CMC-13-5 4,5mM
PROTON.AUT D2O {C:\Bruker\TopSpin3.6.2} AVIII400 8 5
PHA-MAIS-Ruf
CMC-13-4 7,6mM
PROTON.AUT D2O {C:\Bruker\TopSpin3.6.2} AVIII400 9 4
PHA-MAIS-Ruf
CMC-13-3 12,6mM
PROTON.AUT D2O {C:\Bruker\TopSpin3.6.2} AVIII400 10 3
PHA-MAIS-Ruf
CMC-13-2 21mM
PROTON.AUT D2O {C:\Bruker\TopSpin3.6.2} AVIII400 11 2
PHA-MAIS-Ruf
CMC-13-1 35mM
PROTON.AUT D2O {C:\Bruker\TopSpin3.6.2} AVIII400 12 1
f1 (ppm)

**3** + TEA

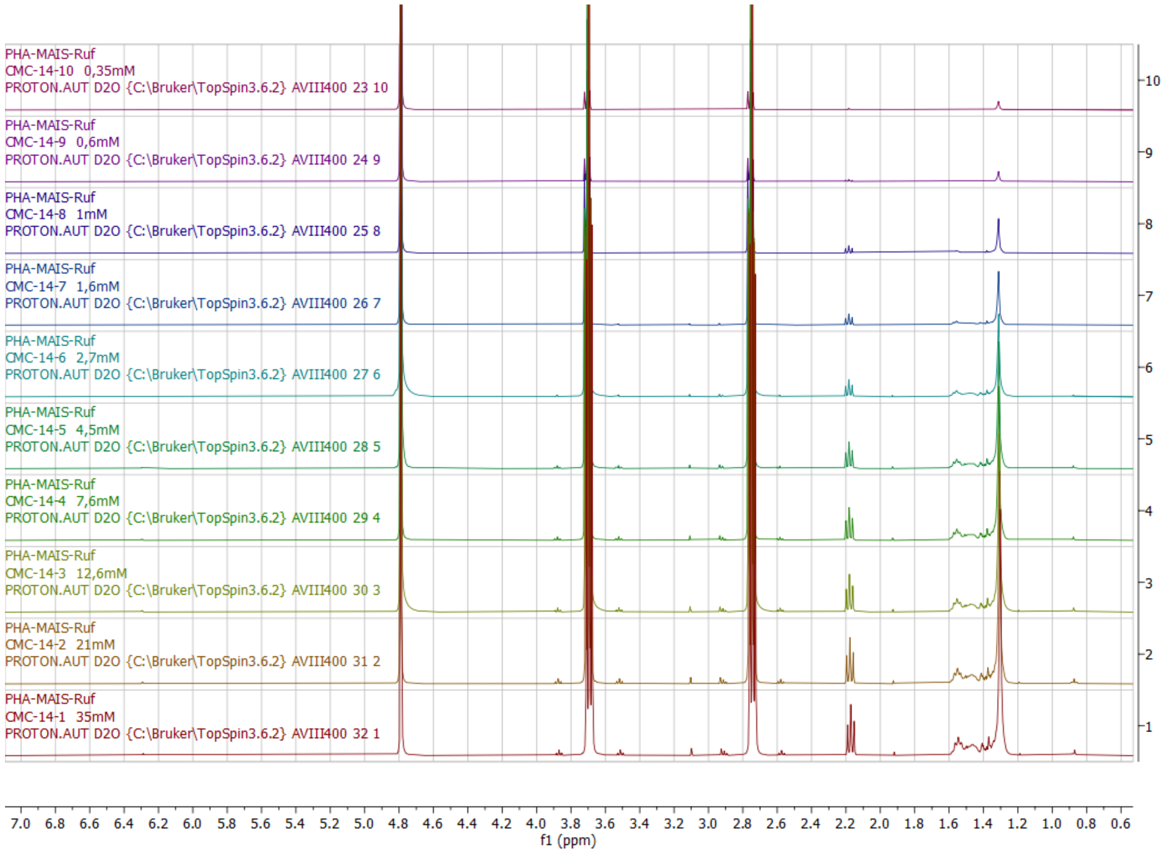
PHA-MAIS-Ruf
CMC-14-10 0,35mM
PROTON.AUT D2O {C:\Bruker\TopSpin3.6.2} AVIII400 23 10
PHA-MAIS-Ruf
CMC-14-9 0,6mM
PROTON.AUT D2O {C:\Bruker\TopSpin3.6.2} AVIII400 24 9
PHA-MAIS-Ruf
CMC-14-8 1mM
PROTON.AUT D2O {C:\Bruker\TopSpin3.6.2} AVIII400 25 8
PHA-MAIS-Ruf
CMC-14-7 1,6mM
PROTON.AUT D2O {C:\Bruker\TopSpin3.6.2} AVIII400 26 7
PHA-MAIS-Ruf
CMC-14-6 2,7mM
PROTON.AUT D2O {C:\Bruker\TopSpin3.6.2} AVIII400 27 6
PHA-MAIS-Ruf
CMC-14-5 4,5mM
PROTON.AUT D2O {C:\Bruker\TopSpin3.6.2} AVIII400 28 5
PHA-MAIS-Ruf
CMC-14-4 7,6mM
PROTON.AUT D2O {C:\Bruker\TopSpin3.6.2} AVIII400 29 4
PHA-MAIS-Ruf
CMC-14-3 12,6mM
PROTON.AUT D2O {C:\Bruker\TopSpin3.6.2} AVIII400 30 3
PHA-MAIS-Ruf
CMC-14-2 21mM
PROTON.AUT D2O {C:\Bruker\TopSpin3.6.2} AVIII400 31 2
PHA-MAIS-Ruf
CMC-14-1 35mM
PROTON.AUT D2O {C:\Bruker\TopSpin3.6.2} AVIII400 32 1
f1 (ppm)

**12**

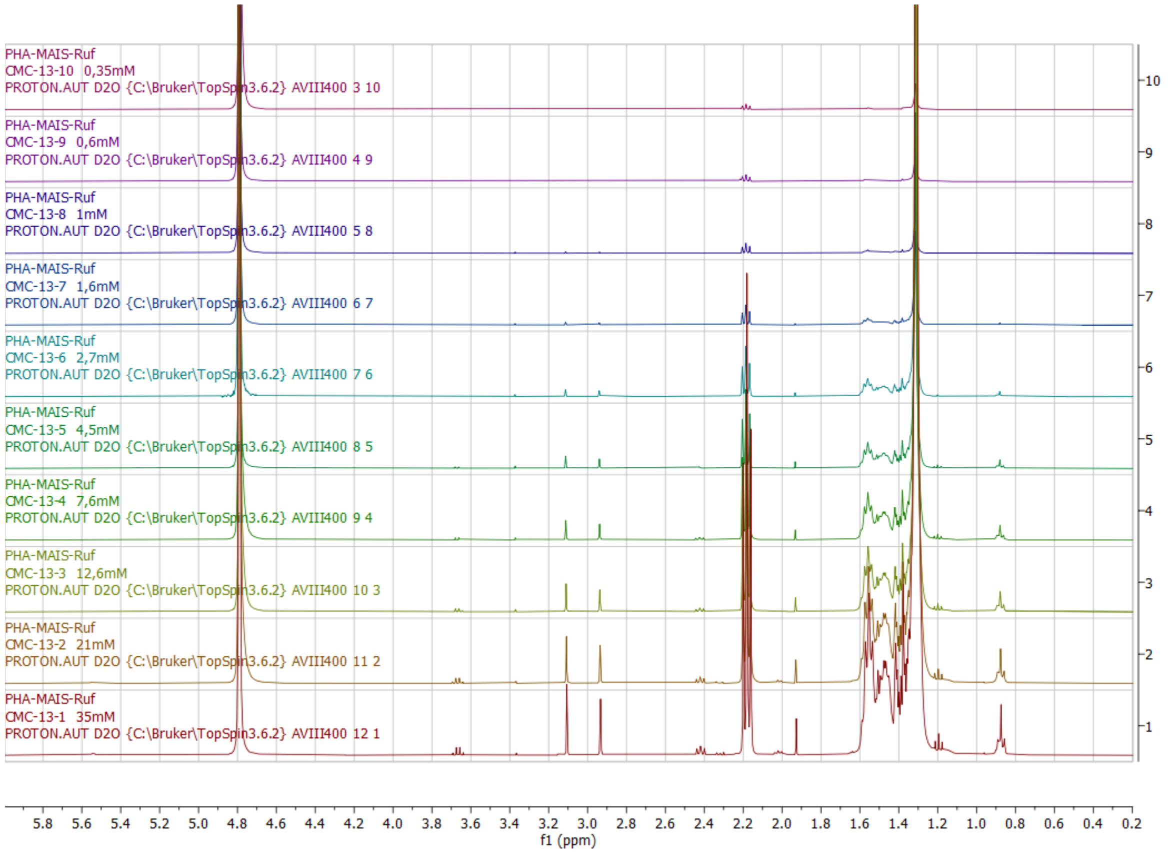

PHA-MAIS-Ruf
CMC-13-10 0,35mM
PROTON.AUT D2O {C:\Bruker\TopSpin3.6.2} AVIII400 3 10
PHA-MAIS-Ruf
CMC-13-9 0,6mM
PROTON.AUT D2O {C:\Bruker\TopSpin3.6.2} AVIII400 4 9
PHA-MAIS-Ruf
CMC-13-8 1mM
PROTON.AUT D2O {C:\Bruker\TopSpin3.6.2} AVIII400 5 8
PHA-MAIS-Ruf
CMC-13-7 1,6mM
PROTON.AUT D2O {C:\Bruker\TopSpin3.6.2} AVIII400 6 7
PHA-MAIS-Ruf
CMC-13-6 2,7mM
PROTON.AUT D2O {C:\Bruker\TopSpin3.6.2} AVIII400 7 6
PHA-MAIS-Ruf
CMC-13-5 4,5mM
PROTON.AUT D2O {C:\Bruker\TopSpin3.6.2} AVIII400 8 5
PHA-MAIS-Ruf
CMC-13-4 7,6mM
PROTON.AUT D2O {C:\Bruker\TopSpin3.6.2} AVIII400 9 4
PHA-MAIS-Ruf
CMC-13-3 12,6mM
PROTON.AUT D2O {C:\Bruker\TopSpin3.6.2} AVIII400 10 3
PHA-MAIS-Ruf
CMC-13-2 21mM
PROTON.AUT D2O {C:\Bruker\TopSpin3.6.2} AVIII400 11 2
PHA-MAIS-Ruf
CMC-13-1 35mM
PROTON.AUT D2O {C:\Bruker\TopSpin3.6.2} AVIII400 12 1
f1 (ppm)


**12** + TEA

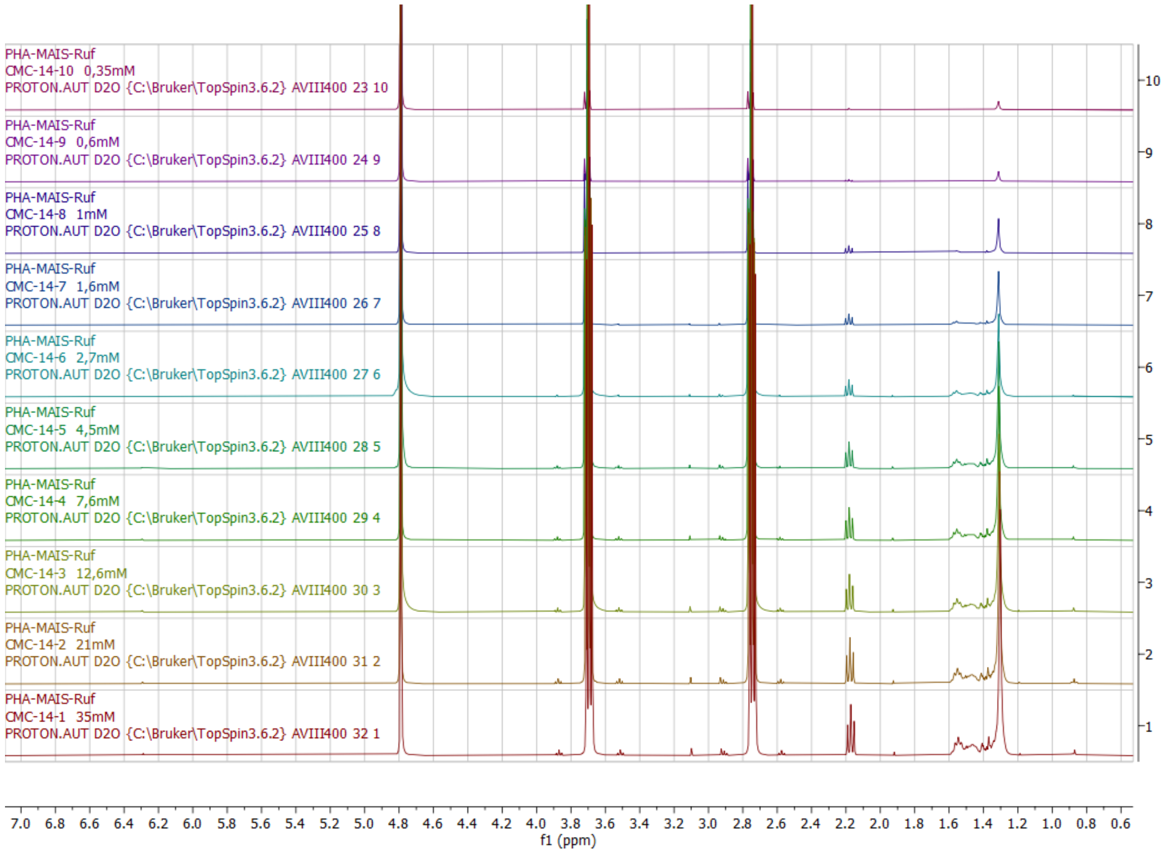

PHA-MAIS-Ruf
CMC-14-10 0,35mM
PROTON.AUT D2O {C:\Bruker\TopSpin3.6.2} AVIII400 23 10
PHA-MAIS-Ruf
CMC-14-9 0,6mM
PROTON.AUT D2O {C:\Bruker\TopSpin3.6.2} AVIII400 24 9
PHA-MAIS-Ruf
CMC-14-8 1mM
PROTON.AUT D2O {C:\Bruker\TopSpin3.6.2} AVIII400 25 8
PHA-MAIS-Ruf
CMC-14-7 1,6mM
PROTON.AUT D2O {C:\Bruker\TopSpin3.6.2} AVIII400 26 7
PHA-MAIS-Ruf
CMC-14-6 2,7mM
PROTON.AUT D2O {C:\Bruker\TopSpin3.6.2} AVIII400 27 6
PHA-MAIS-Ruf
CMC-14-5 4,5mM
PROTON.AUT D2O {C:\Bruker\TopSpin3.6.2} AVIII400 28 5
PHA-MAIS-Ruf
CMC-14-4 7,6mM
PROTON.AUT D2O {C:\Bruker\TopSpin3.6.2} AVIII400 29 4
PHA-MAIS-Ruf
CMC-14-3 12,6mM
PROTON.AUT D2O {C:\Bruker\TopSpin3.6.2} AVIII400 30 3
PHA-MAIS-Ruf
CMC-14-2 21mM
PROTON.AUT D2O {C:\Bruker\TopSpin3.6.2} AVIII400 31 2
PHA-MAIS-Ruf
CMC-14-1 35mM
PROTON.AUT D2O {C:\Bruker\TopSpin3.6.2} AVIII400 32 1
f1 (ppm)

**18**

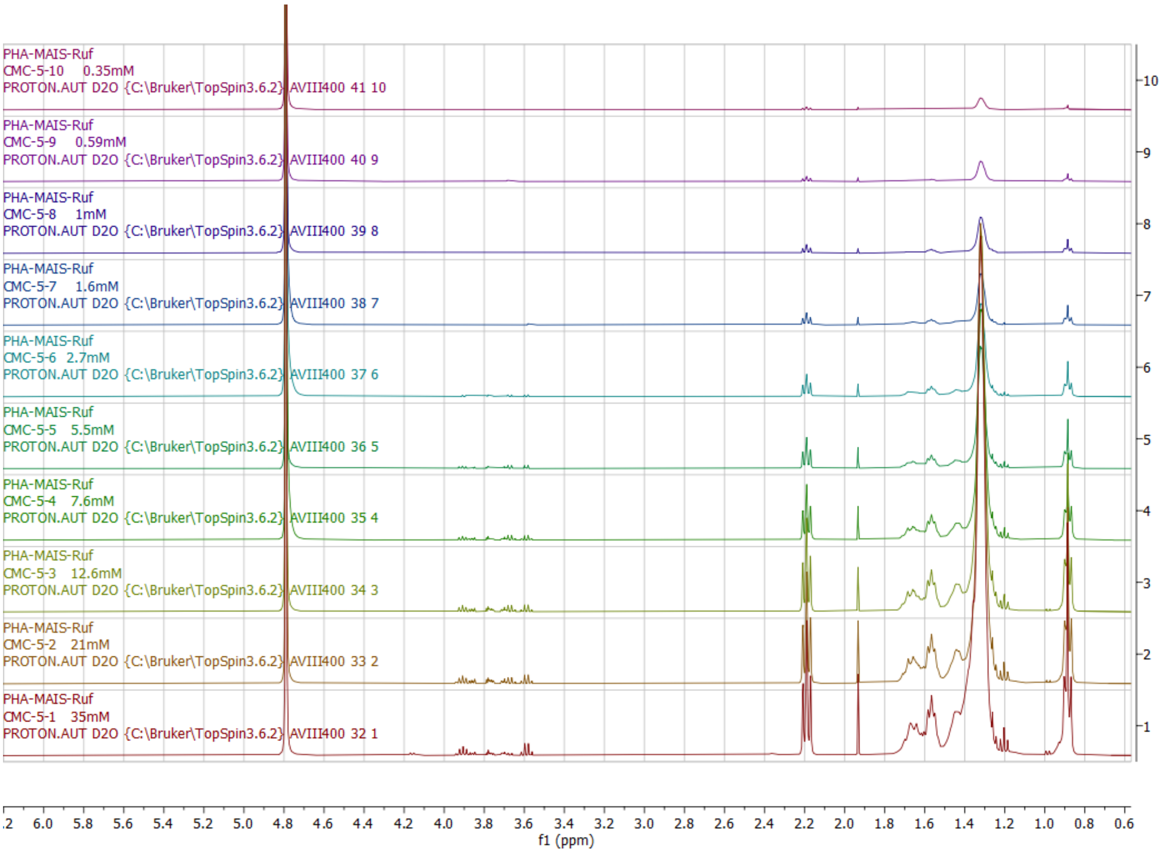
PHA-MAIS-Ruf
CMC-5-10 0.35mM
PROTON.AUT D2O {C:\Bruker\TopSpin3.6.2} AVIII400 41 10
PHA-MAIS-Ruf
CMC-5-9 0.59mM
PROTON.AUT D2O {C:\Bruker\TopSpin3.6.2} AVIII400 40 9
PHA-MAIS-Ruf
CMC-5-8 1mM
PROTON.AUT D2O {C:\Bruker\TopSpin3.6.2} AVIII400 39 8
PHA-MAIS-Ruf
CMC-5-7 1.6mM
PROTON.AUT D2O {C:\Bruker\TopSpin3.6.2} AVIII400 38 7
PHA-MAIS-Ruf
CMC-5-6 2.7mM
PROTON.AUT D2O {C:\Bruker\TopSpin3.6.2} AVIII400 37 6
PHA-MAIS-Ruf
CMC-5-5 5.5mM
PROTON.AUT D2O {C:\Bruker\TopSpin3.6.2} AVIII400 36 5
PHA-MAIS-Ruf
CMC-5-4 7.6mM
PROTON.AUT D2O {C:\Bruker\TopSpin3.6.2} AVIII400 35 4
PHA-MAIS-Ruf
CMC-5-3 12.6mM
PROTON.AUT D2O {C:\Bruker\TopSpin3.6.2} AVIII400 34 3
PHA-MAIS-Ruf
CMC-5-2 21mM
PROTON.AUT D2O {C:\Bruker\TopSpin3.6.2} AVIII400 33 2
PHA-MAIS-Ruf
CMC-5-1 35mM
PROTON.AUT D2O {C:\Bruker\TopSpin3.6.2} AVIII400 32 1
f1 (ppm)

**18** + TEA

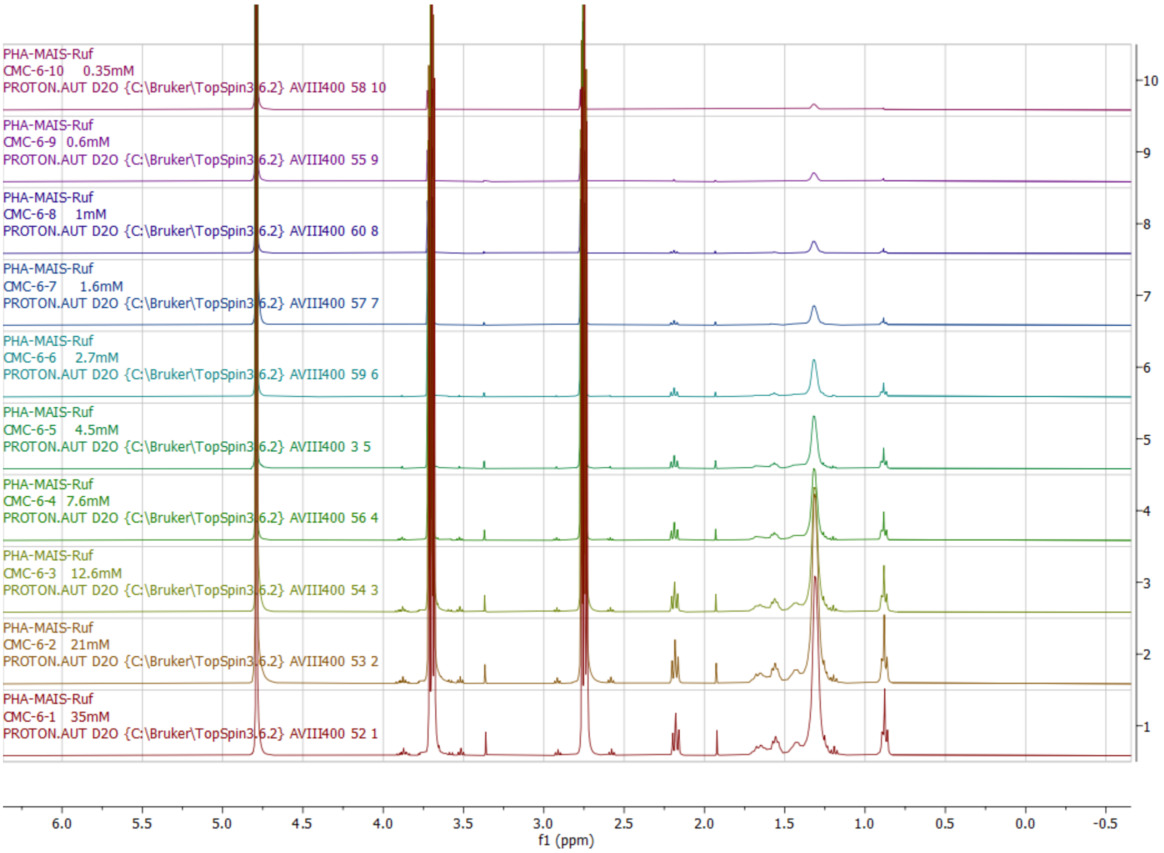
PHA-MAIS-Ruf
CMC-6-10 0.35mM
PROTON.AUT D2O {C:\Bruker\TopSpin3.6.2} AVIII400 58 10
PHA-MAIS-Ruf
CMC-6-9 0.6mM
PROTON.AUT D2O {C:\Bruker\TopSpin3.6.2} AVIII400 55 9
PHA-MAIS-Ruf
CMC-6-8 1mM
PROTON.AUT D2O {C:\Bruker\TopSpin3.6.2} AVIII400 60 8
PHA-MAIS-Ruf
CMC-6-7 1.6mM
PROTON.AUT D2O {C:\Bruker\TopSpin3.6.2} AVIII400 57 7
PHA-MAIS-Ruf
CMC-6-6 2.7mM
PROTON.AUT D2O {C:\Bruker\TopSpin3.6.2} AVIII400 59 6
PHA-MAIS-Ruf
CMC-6-5 4.5mM
PROTON.AUT D2O {C:\Bruker\TopSpin3.6.2} AVIII400 3 5
PHA-MAIS-Ruf
CMC-6-4 7.6mM
PROTON.AUT D2O {C:\Bruker\TopSpin3.6.2} AVIII400 56 4
PHA-MAIS-Ruf
CMC-6-3 12.6mM
PROTON.AUT D2O {C:\Bruker\TopSpin3.6.2} AVIII400 54 3
PHA-MAIS-Ruf
CMC-6-2 21mM
PROTON.AUT D2O {C:\Bruker\TopSpin3.6.2} AVIII400 53 2
PHA-MAIS-Ruf
CMC-6-1 35mM
PROTON.AUT D2O {C:\Bruker\TopSpin3.6.2} AVIII400 52 1
f1 (ppm)

# NMR-Spectra

## 10-Carboxyundecyl phosphonic acid 3

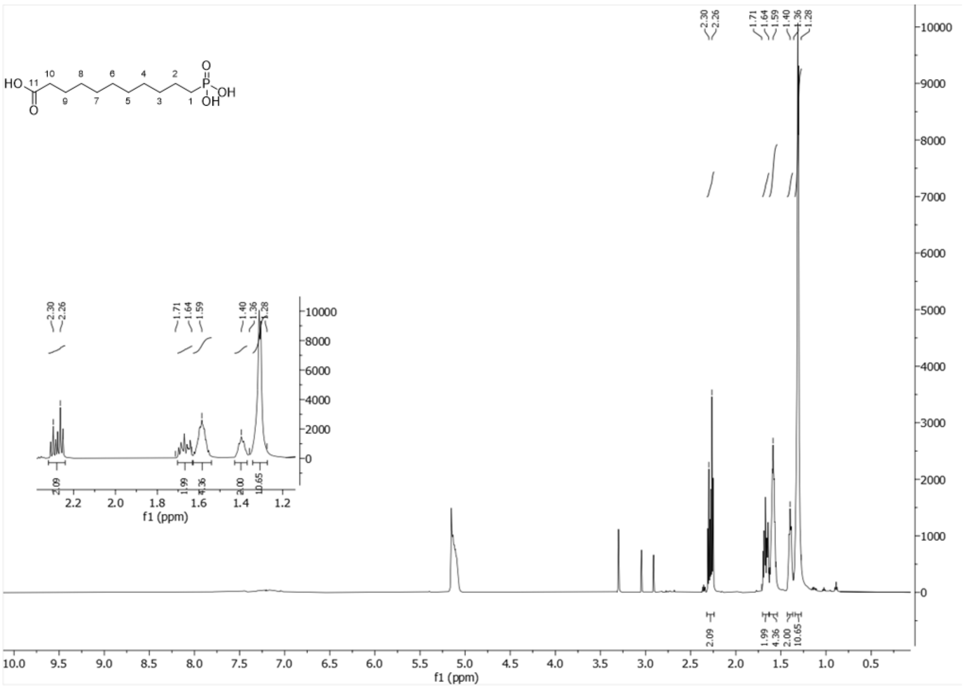


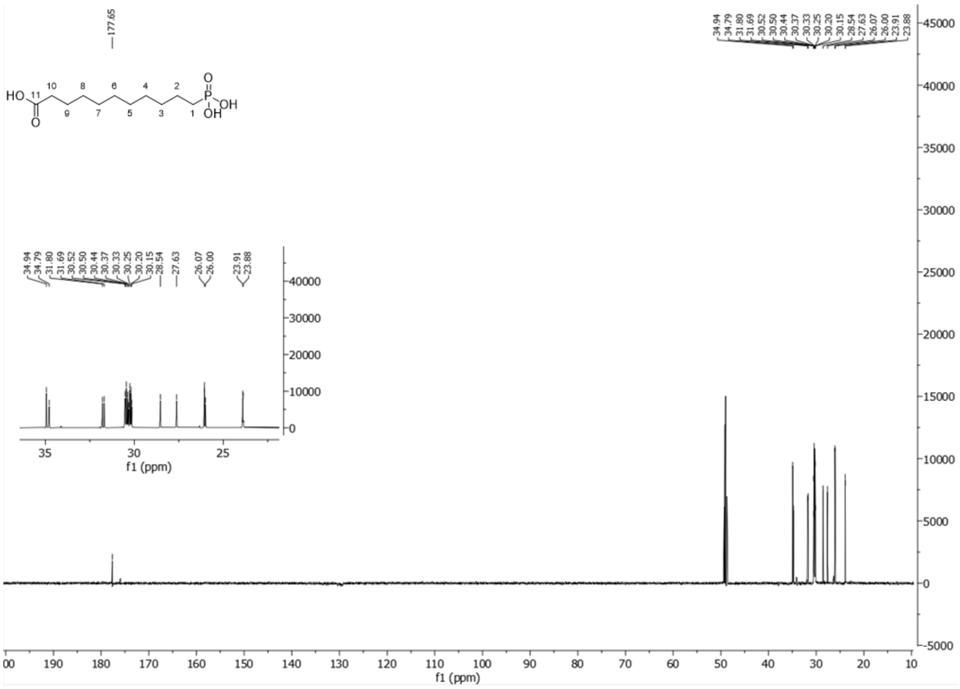

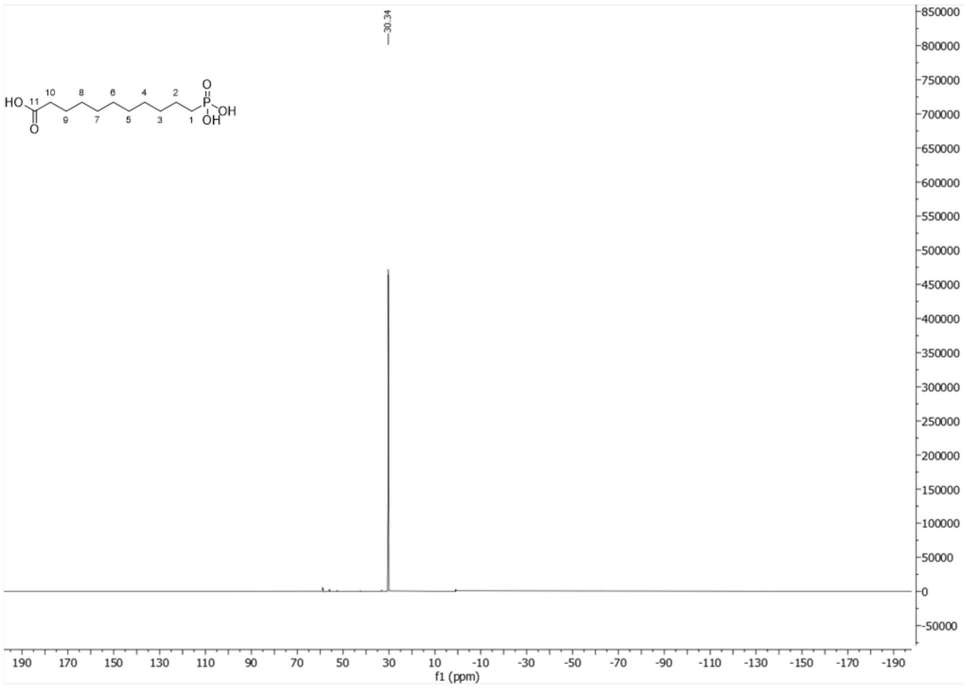

30.34
HO
11
10
9
8
7
6
5
4
3
2
1
O
P
OH
OH
f1 (ppm)

**(E)-(3,7-dimethylocta-2,6-dien-1-yl)phosphonic acid 6**

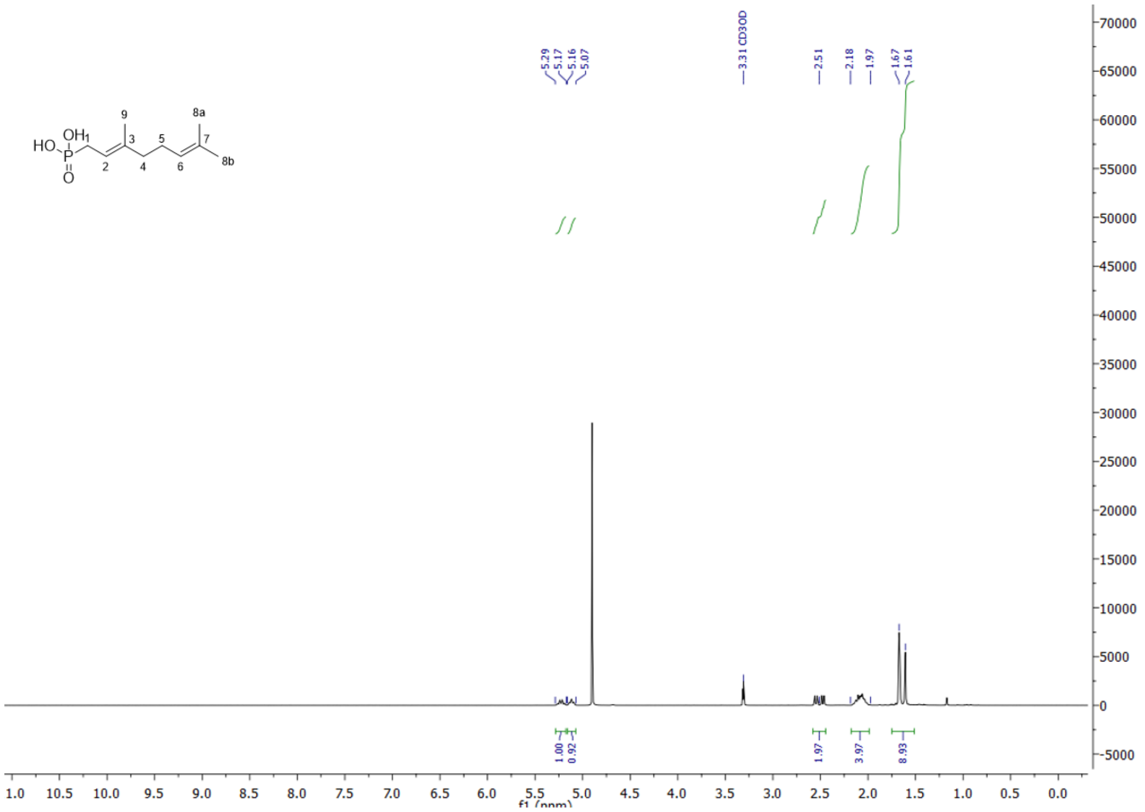

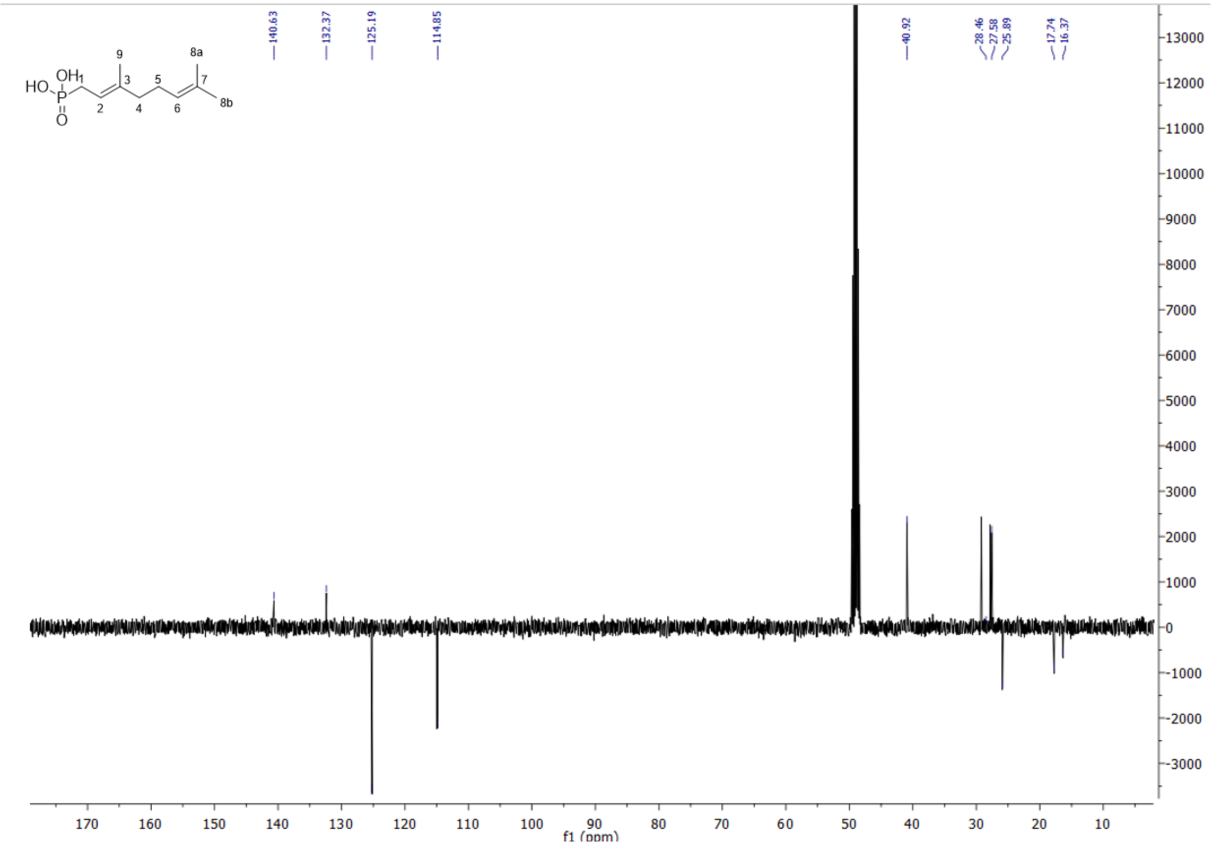


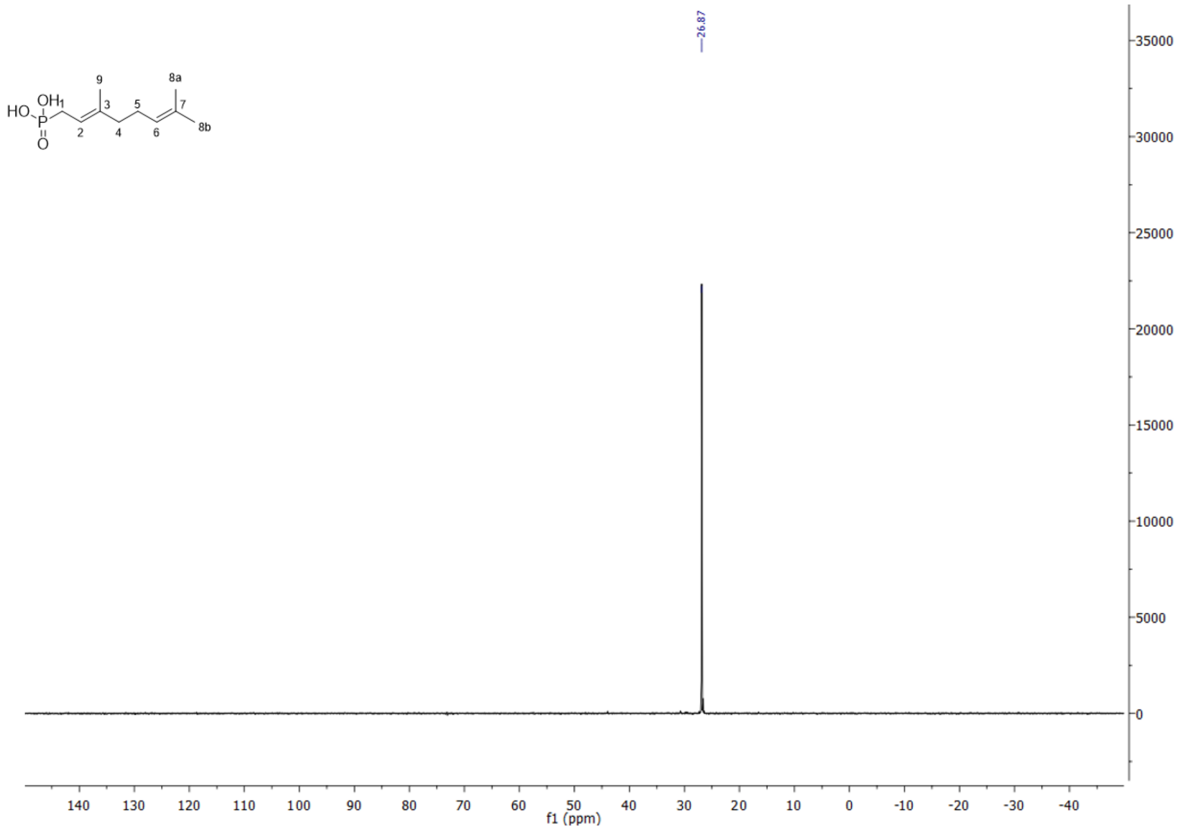


**((3,3-Dimethylbicyclo[2.2.1]heptan-2-yl)methyl)phosphonic acid 7**

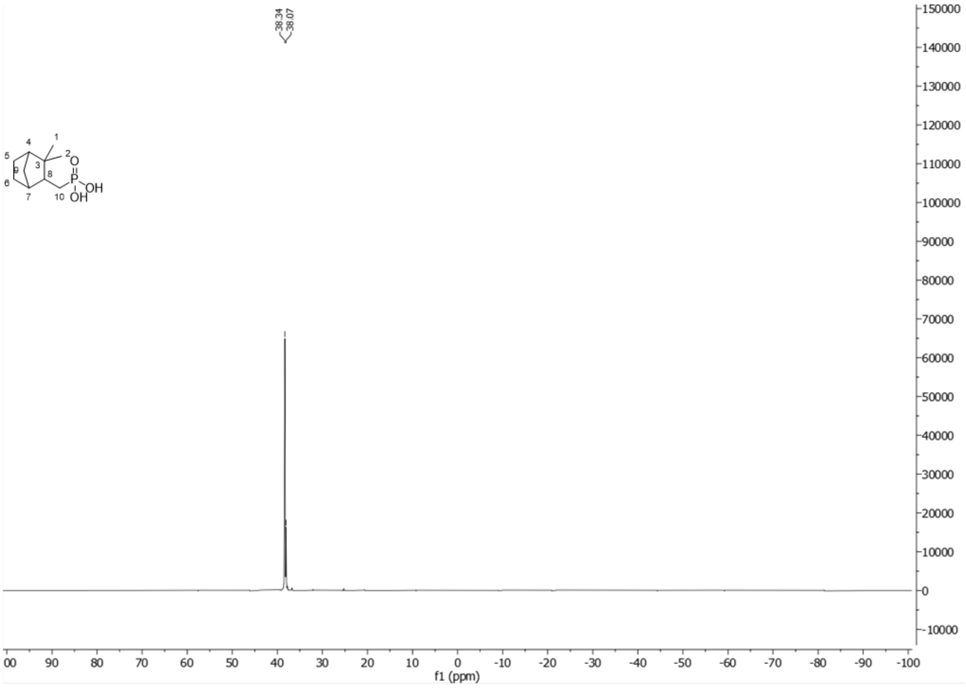
38.34
38.07
f1 (ppm)
OH
OH
O
P

**(2-(2-hydroxy-4-methylcyclohexyl)propyl)phosphonic acid 8**

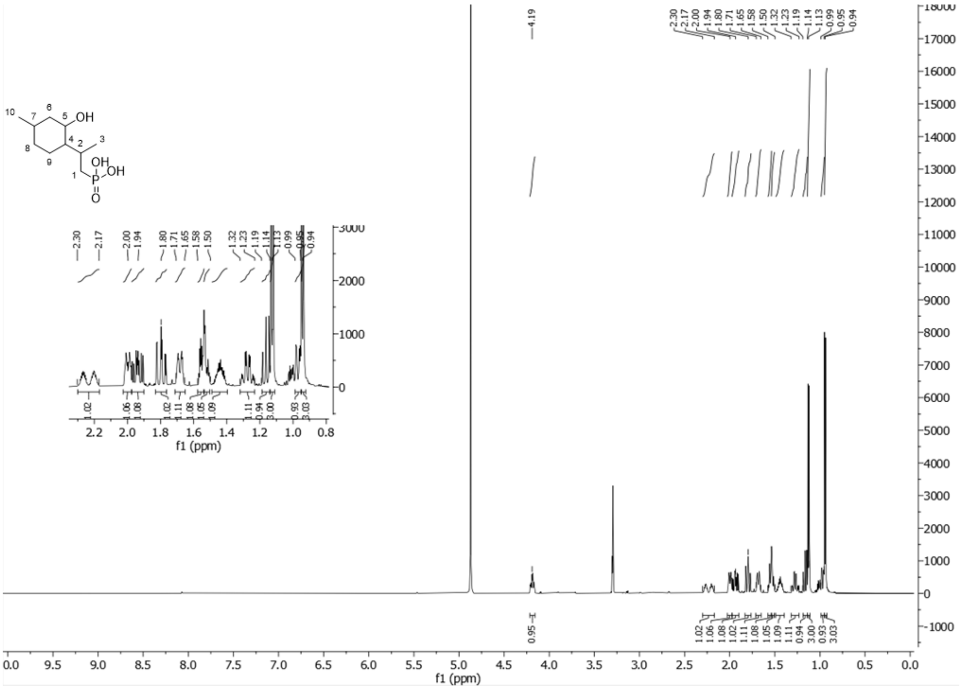

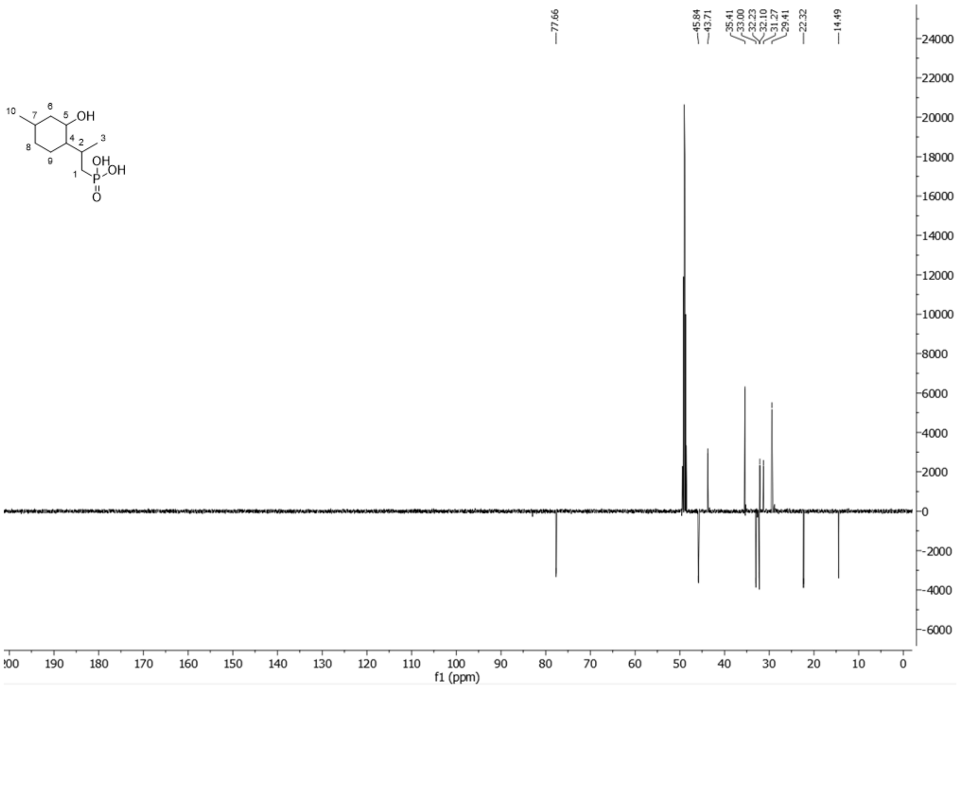

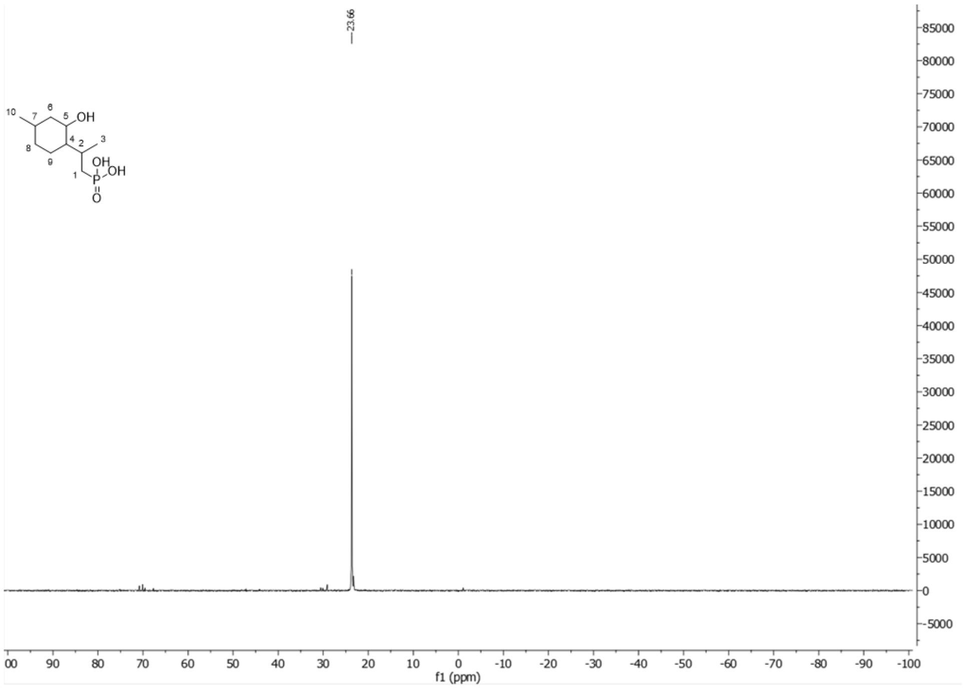
23.66
10
7
6
5
OH
8
4
2
3
9
1
OH
P
OH
O
f1 (ppm)

## ((6,6-Dimethylbicyclo[3.1.1]heptan2-yl)methyl)phosphonic acid 9

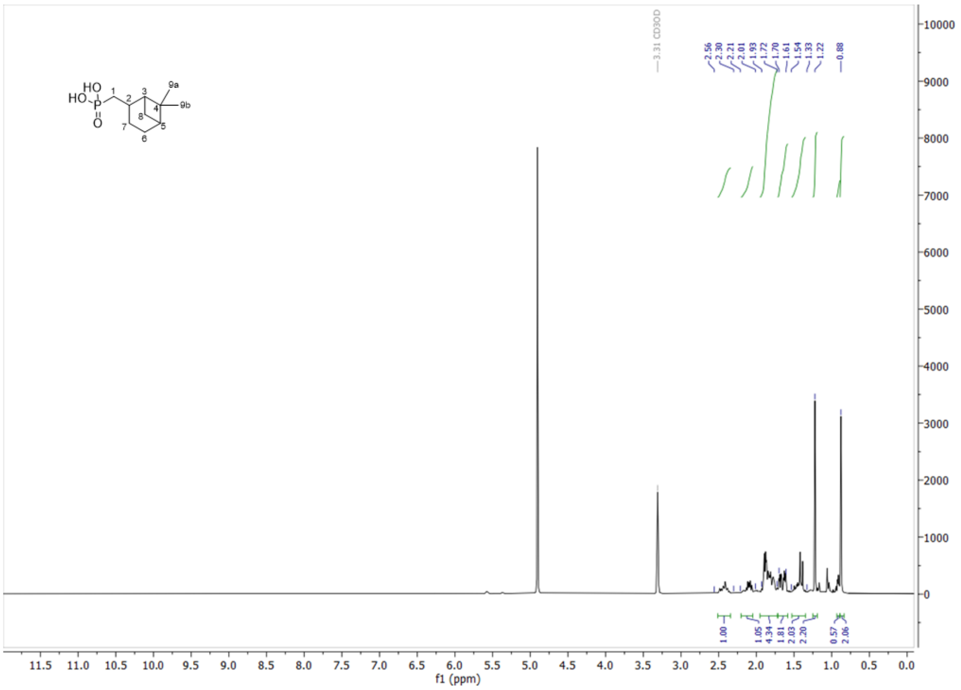

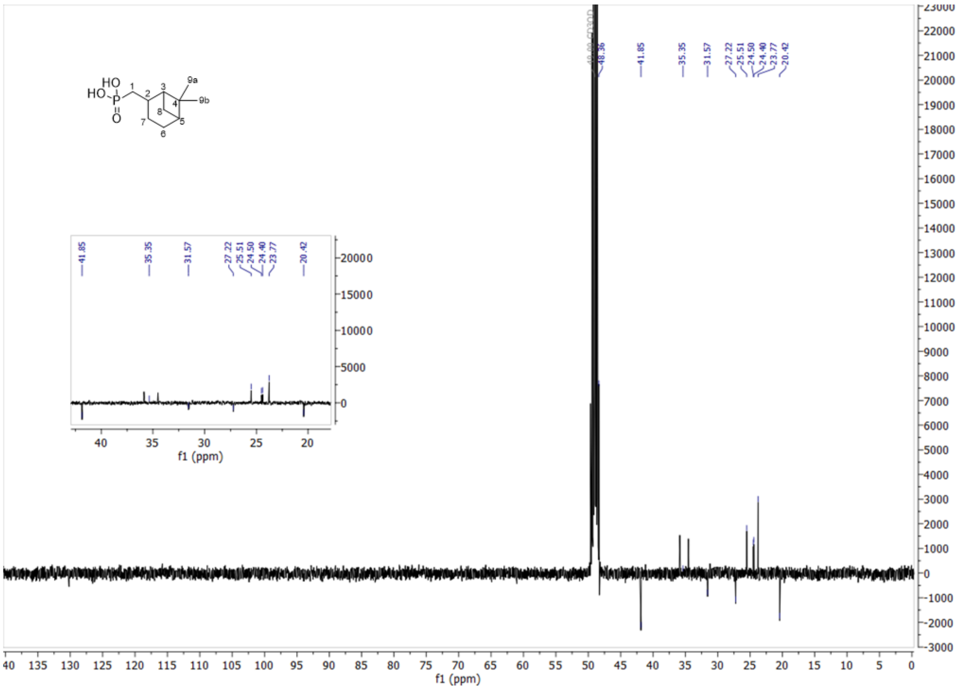

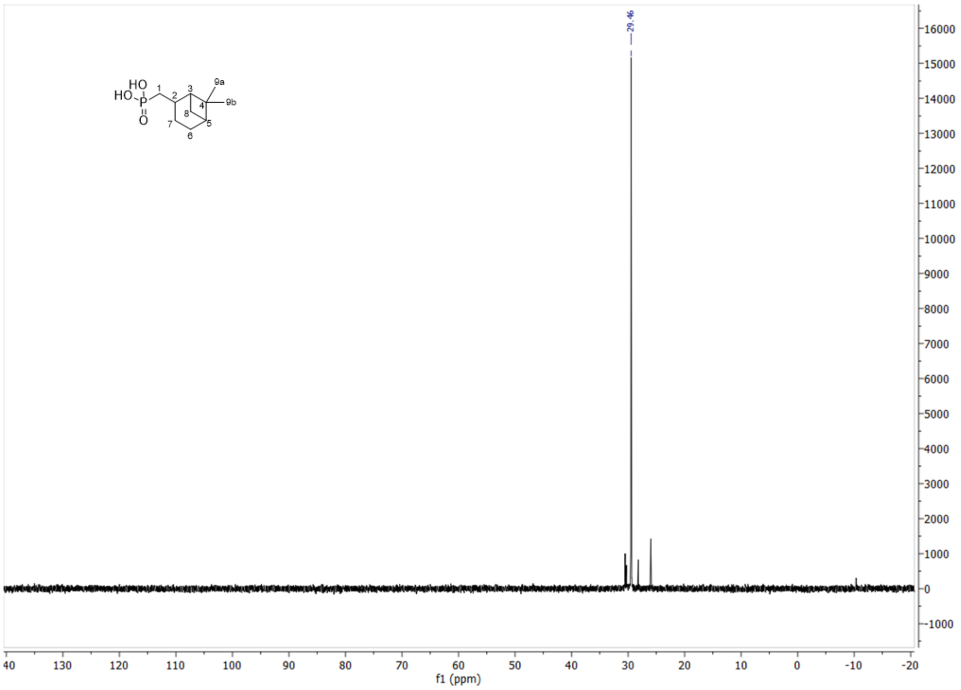

29.46
HO
HO
P
O
1
2
3
4
5
6
7
8
9a
9b
16000
15000
14000
13000
12000
11000
10000
9000
8000
7000
6000
5000
4000
3000
2000
1000
0
-1000
40
130
120
110
100
90
80
70
60
50
40
30
20
10
0
-10
-20
f1 (ppm)

## ((4-Isopropylcyclohex-1-en-1-yl)methyl)phosphonic acid 12

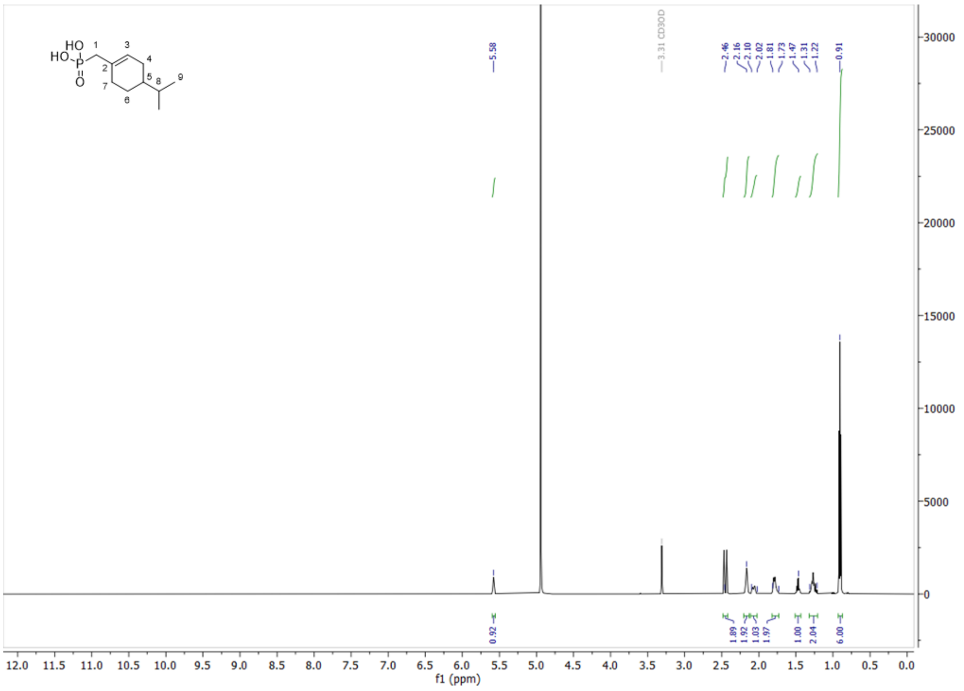

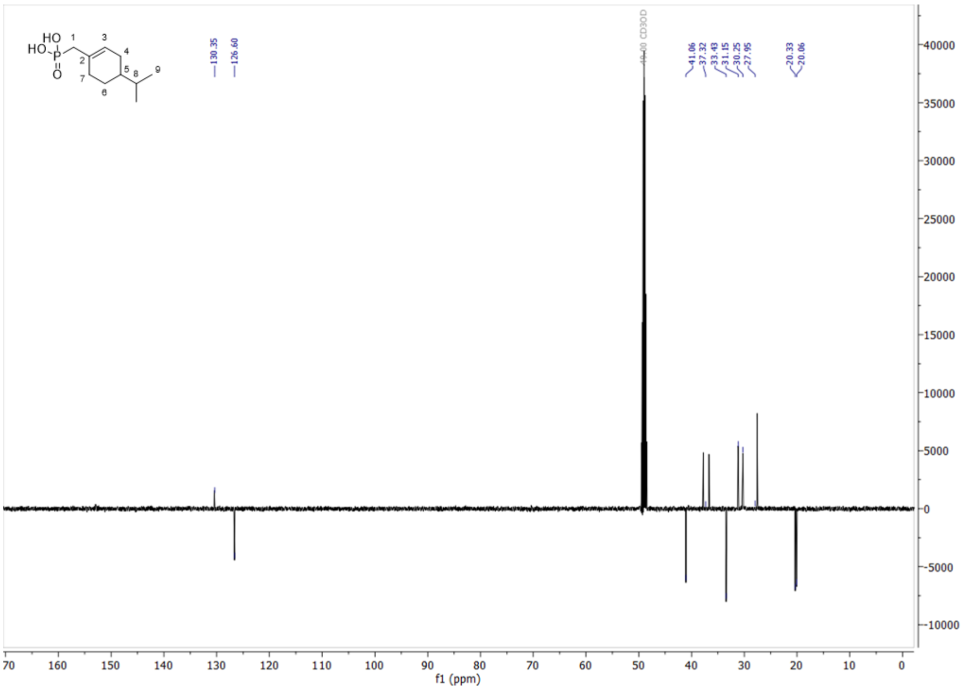

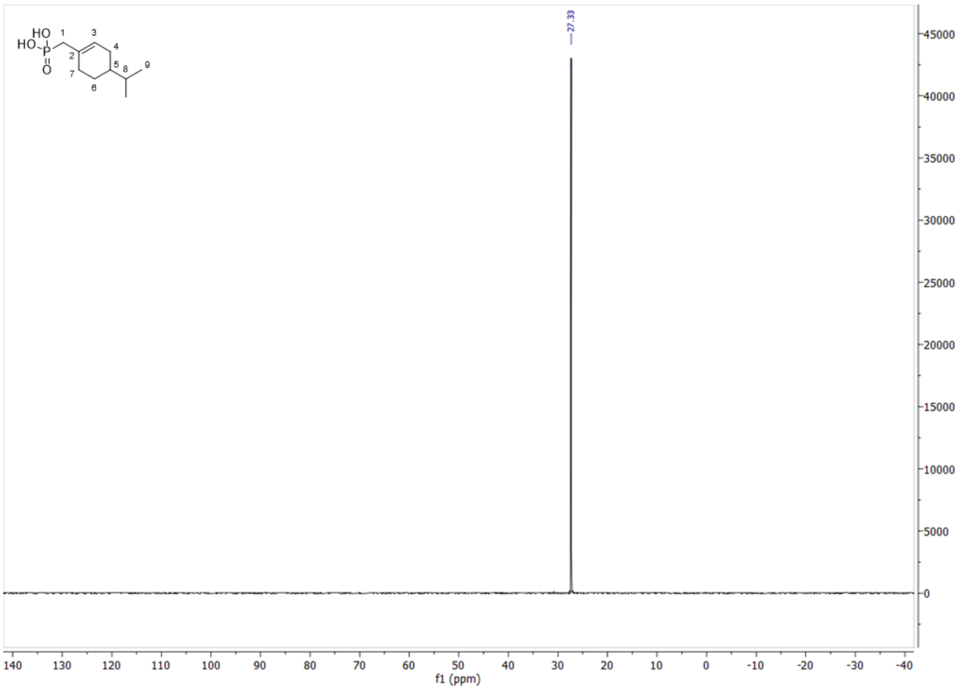

HO
HO
P
O
1
2
3
4
5
6
7
8
9
27.33
45000
40000
35000
30000
25000
20000
15000
10000
5000
0
140
130
120
110
100
90
80
70
60
50
40
30
20
10
0
-10
-20
-30
-40
f1 (ppm)

**(5-(1-(diethoxyphosphoryl)propan-2-yl)-2-methylcyclohexyl)phosphonate**

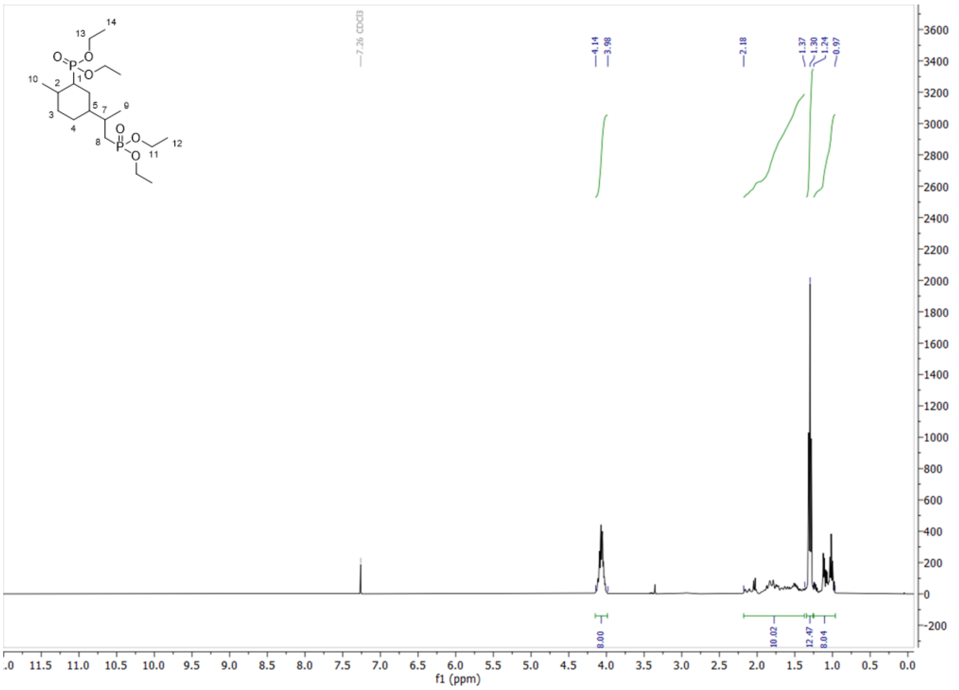


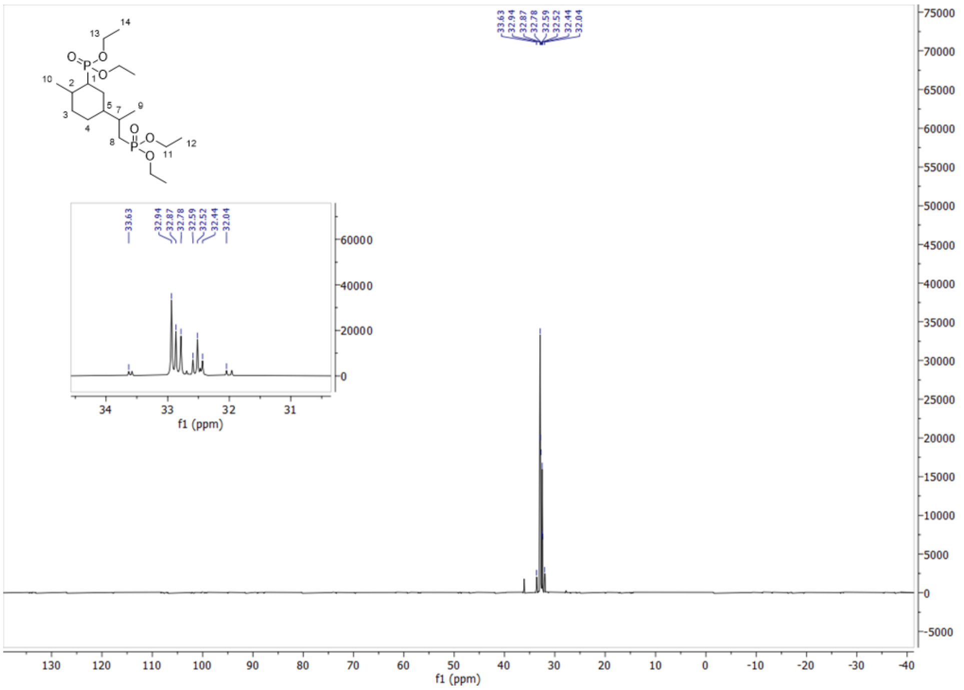

**(2-Methyl-5-(1-phosphonopropan-2-yl)cyclohexyl)phosphonic acid 14**

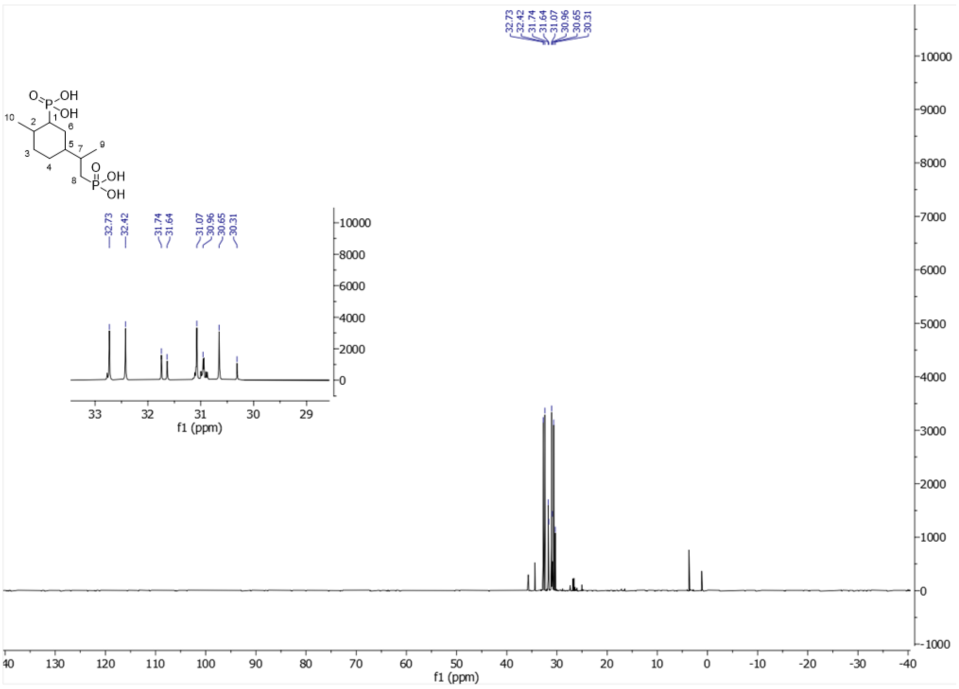

**Diethyl ((2-(diethoxyphosphoryl)-4-isopropylcyclohexyl)methyl)phosphonate**

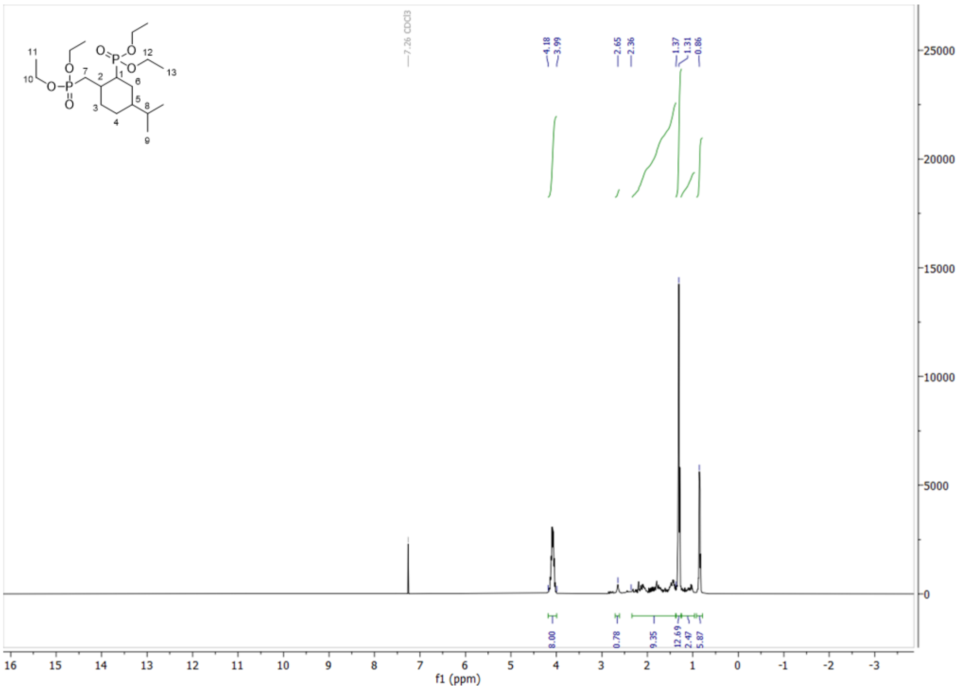


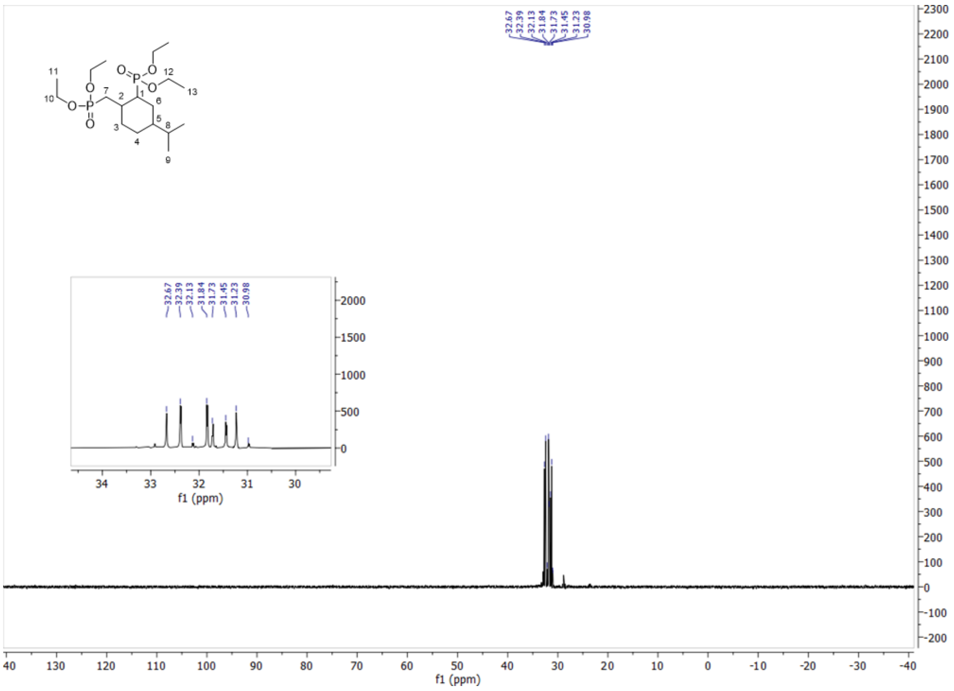

**((4-Isopropyl-2-phosphonocyclohexyl)methyl)phosphonic acid 15**

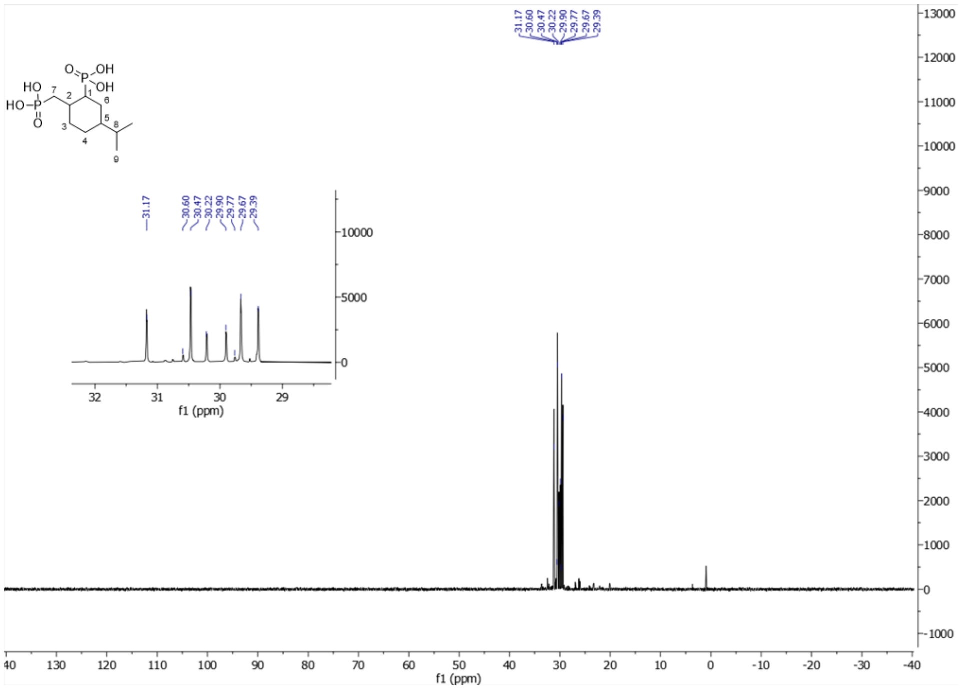

**(6-(1-(diethoxyphosphoryl)propan-2-yl)-4,4a-dimethyldecahydronaphthalen-1-yl)phosphonate**

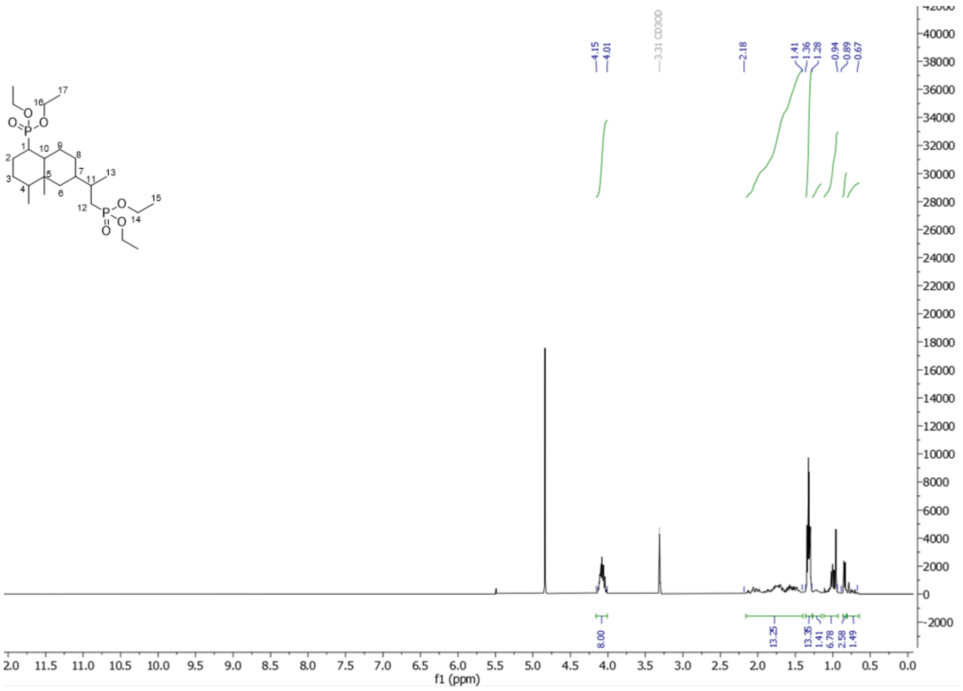


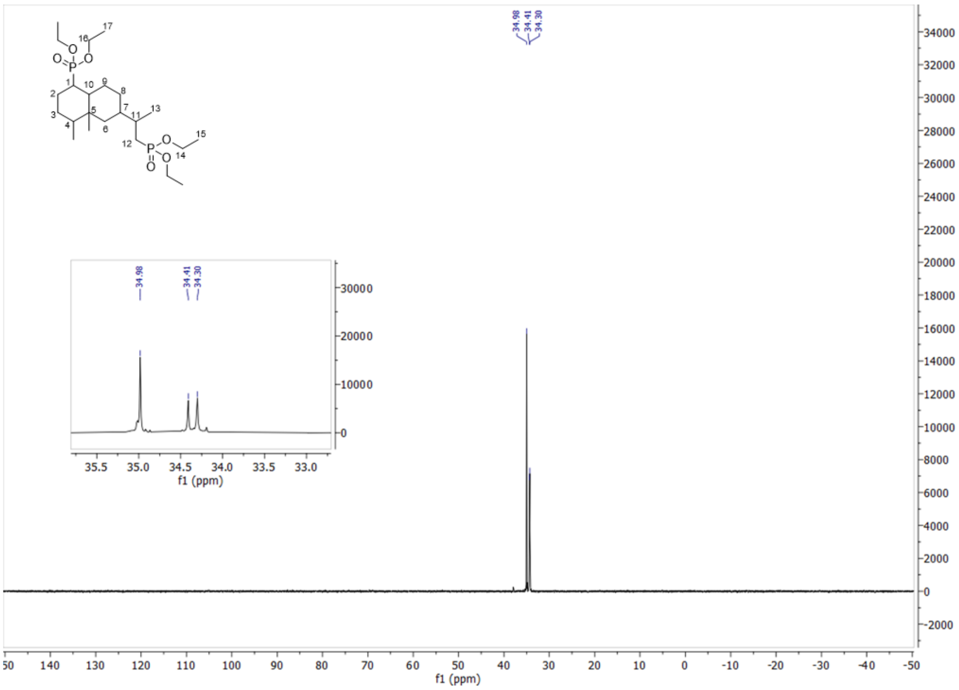

**(4,4a-Dimethyl-6-(1-phosphonopropan-2-yl)decahydronaphthalen-1-yl)phosphonic acid 16**

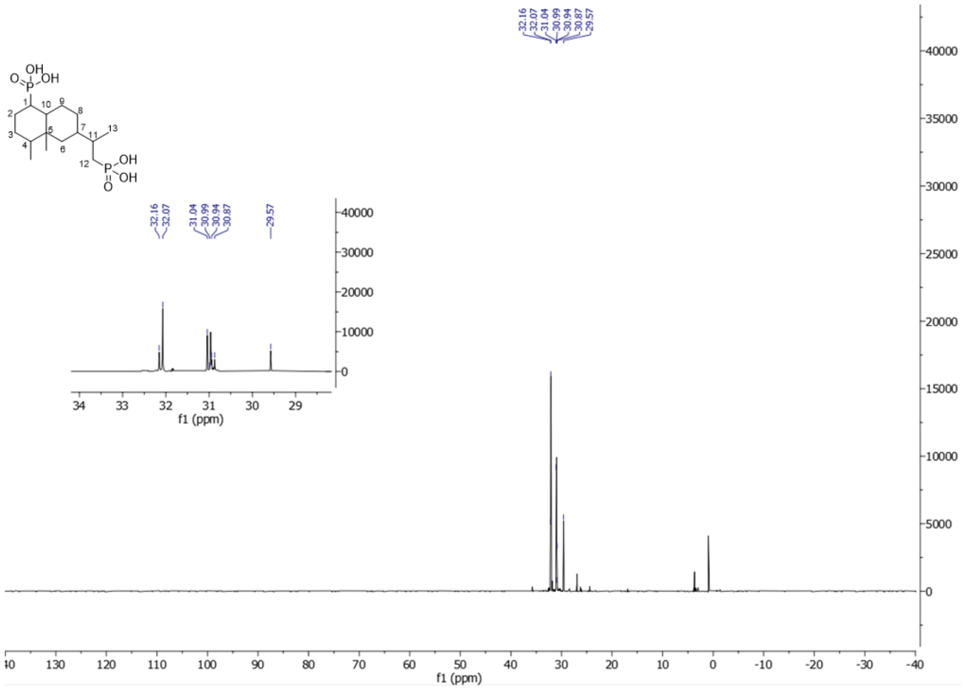

# 10-(diethoxyphosphoryl)octadecanoic acid

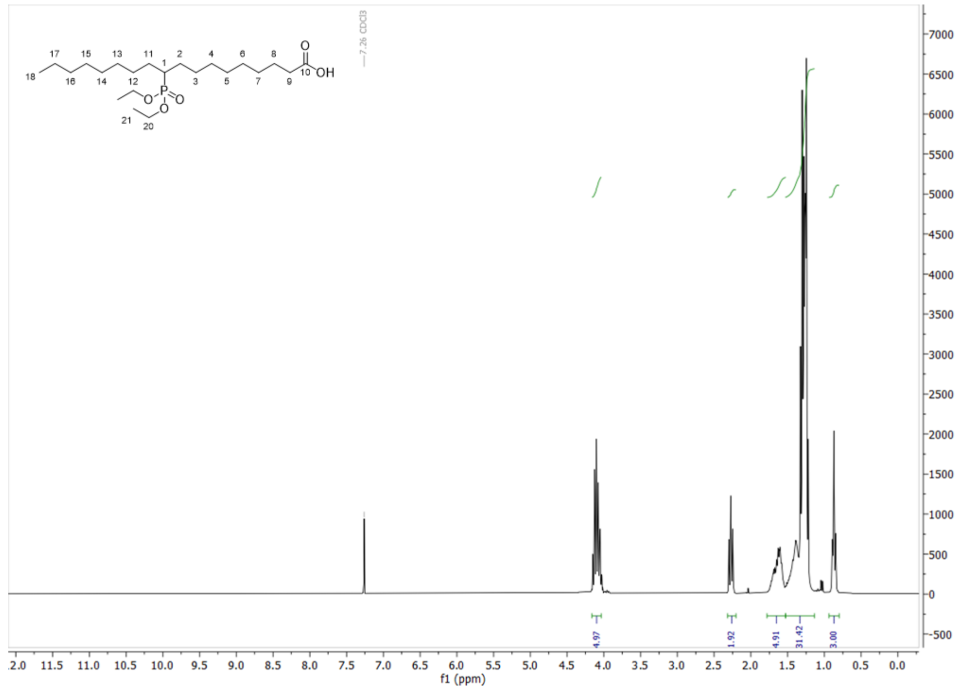


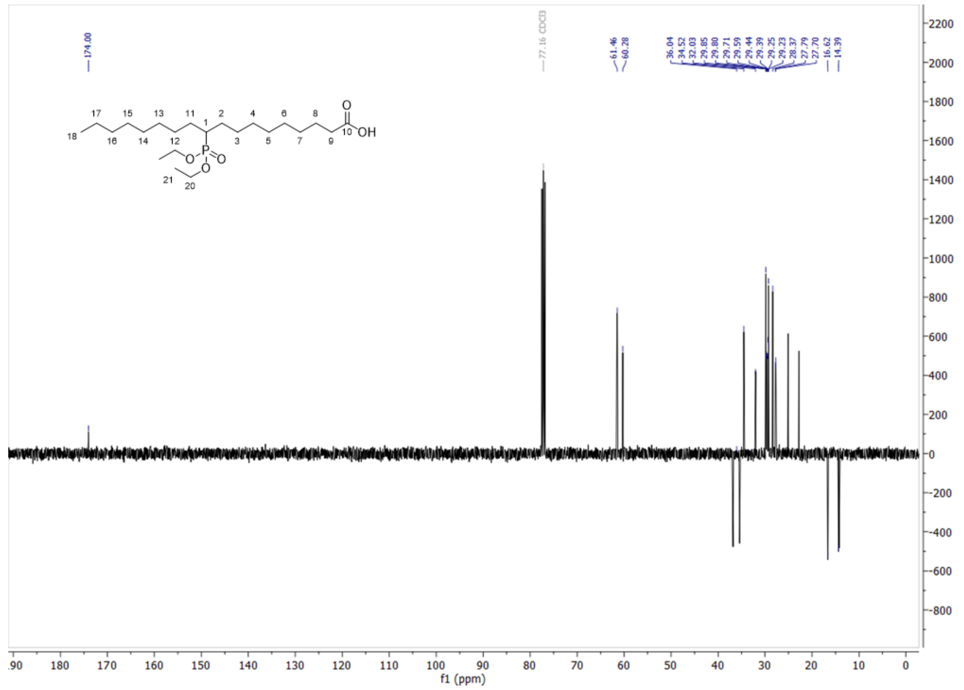

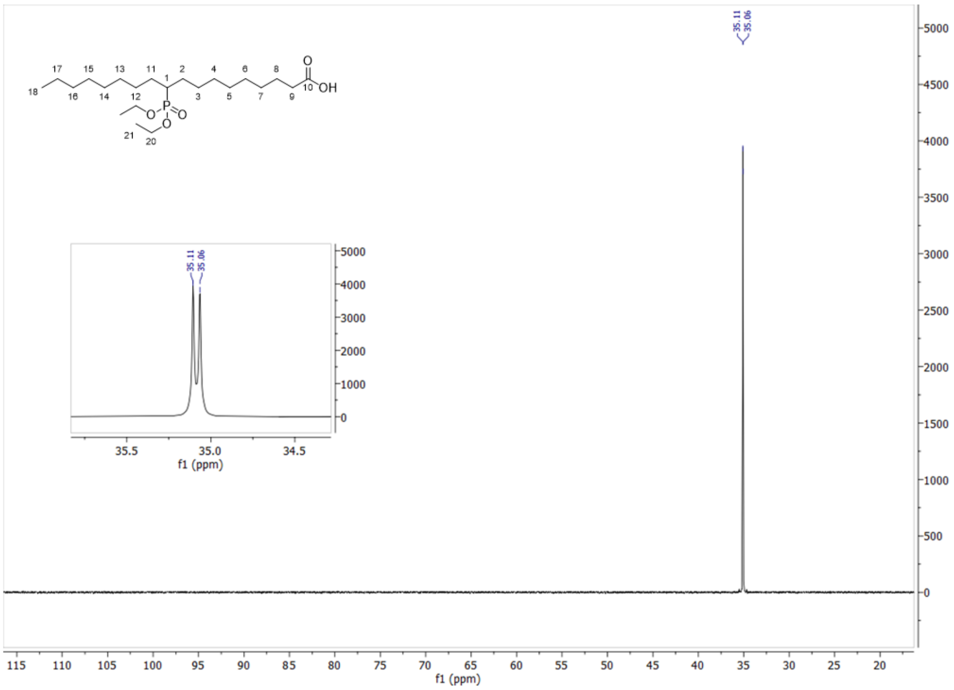
35.11
35.06
f1 (ppm)
OH

## 10-Phosphonooctadecanoic acid 18

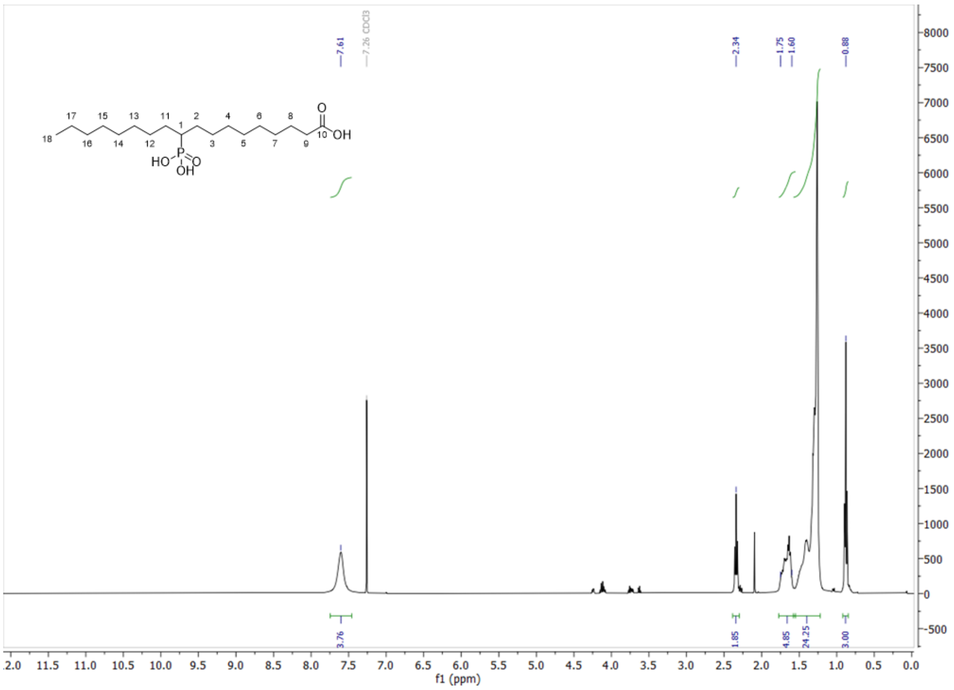

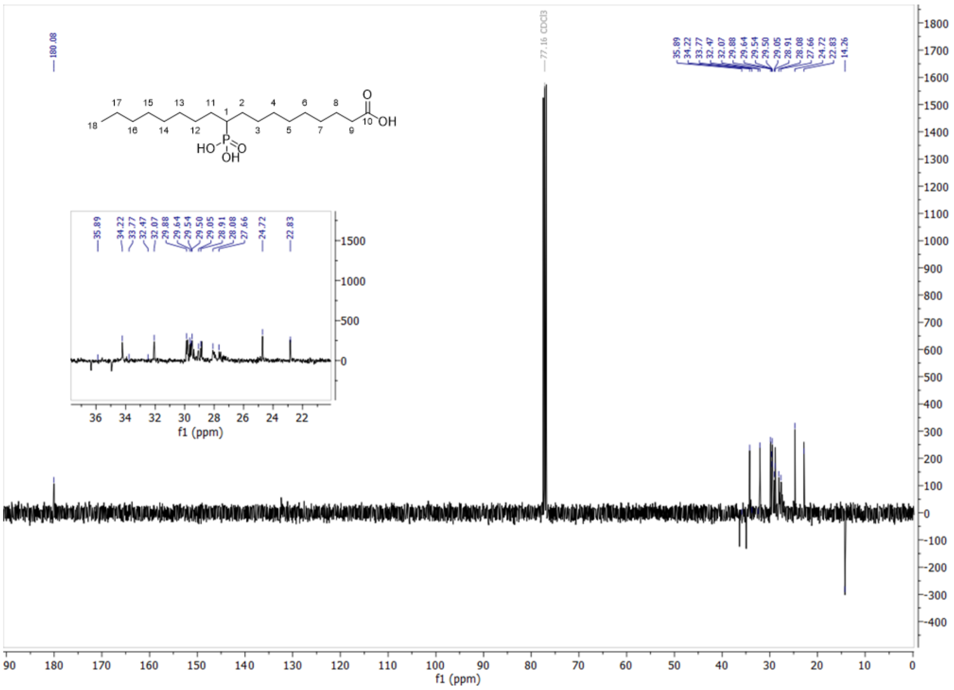

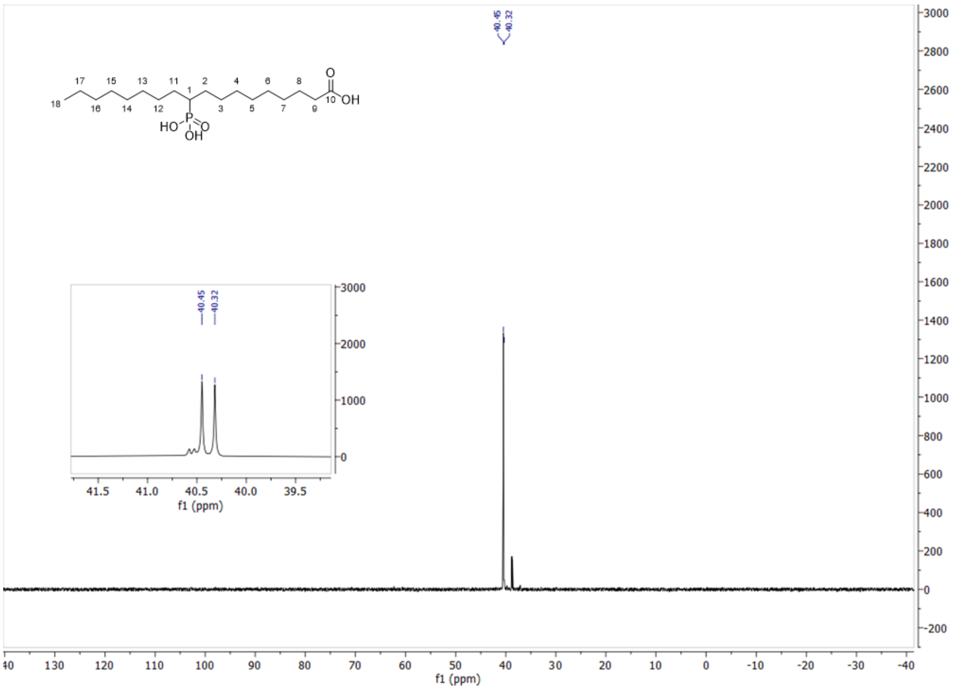
40.45
40.32
HO
OH
OH
f1 (ppm)

**10-(Diethoxyphosphoryl)octadec-9-enoic acid 23**

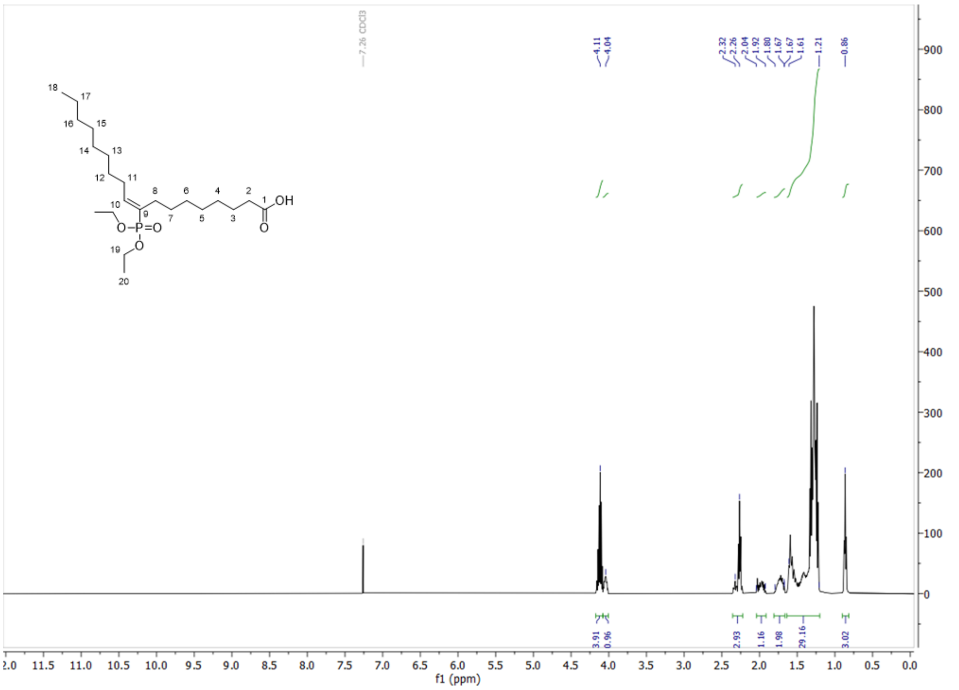


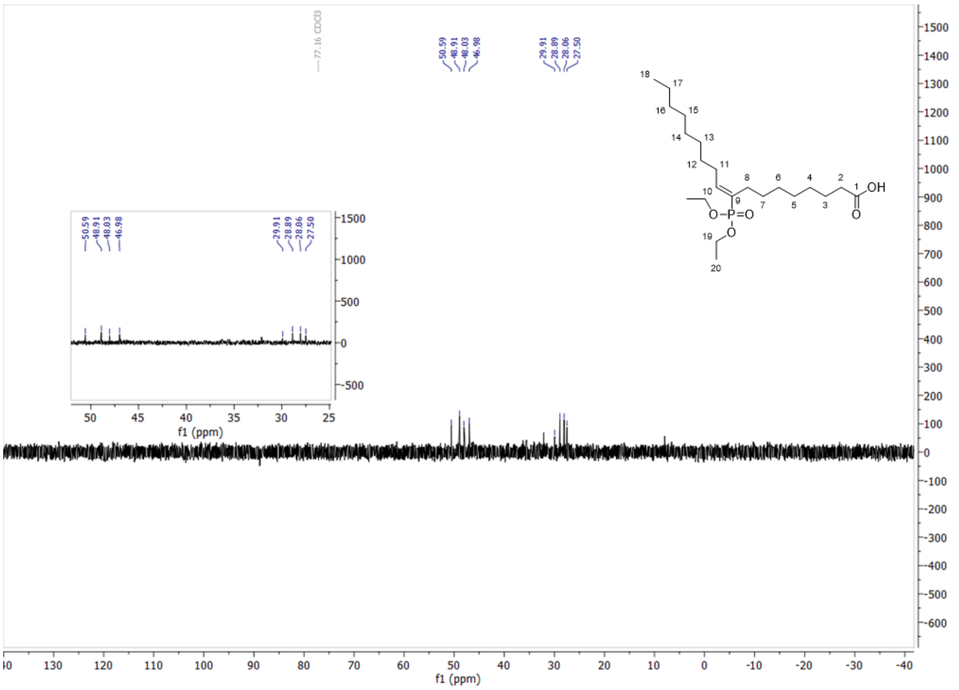

**10-Phosphonooctadec-9-enoic acid 24**

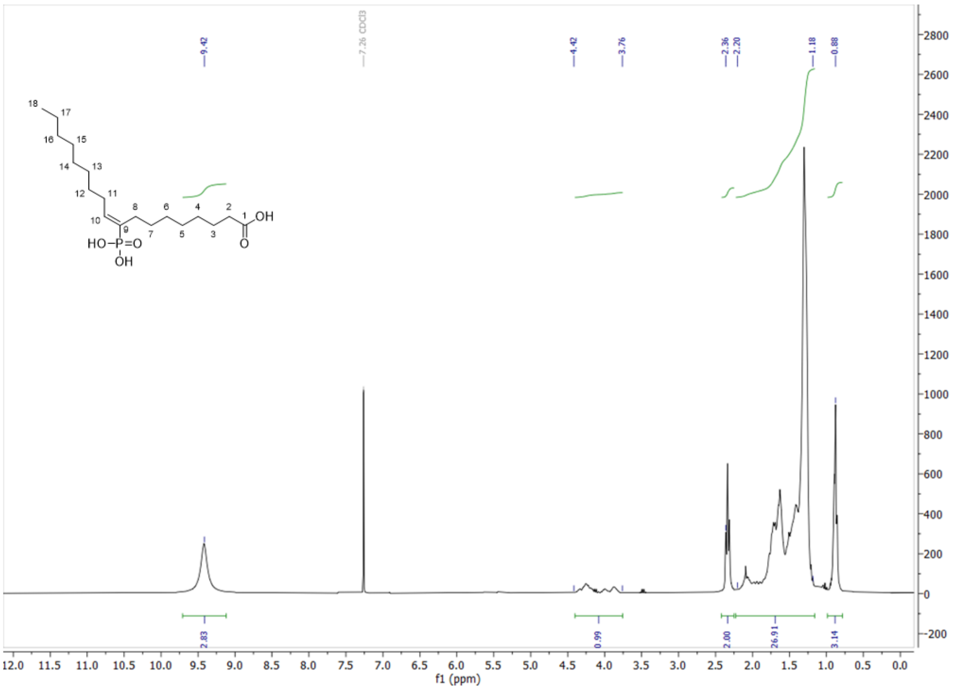


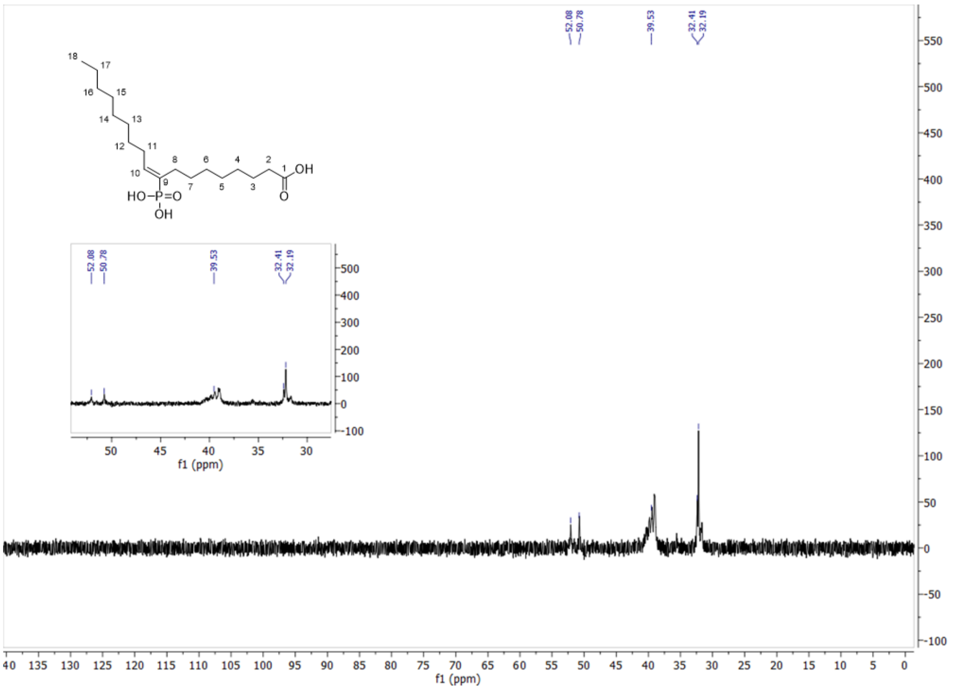

**Diethyl (3,7-dimethyloct-6-en-1-yl)phosphonate 26**

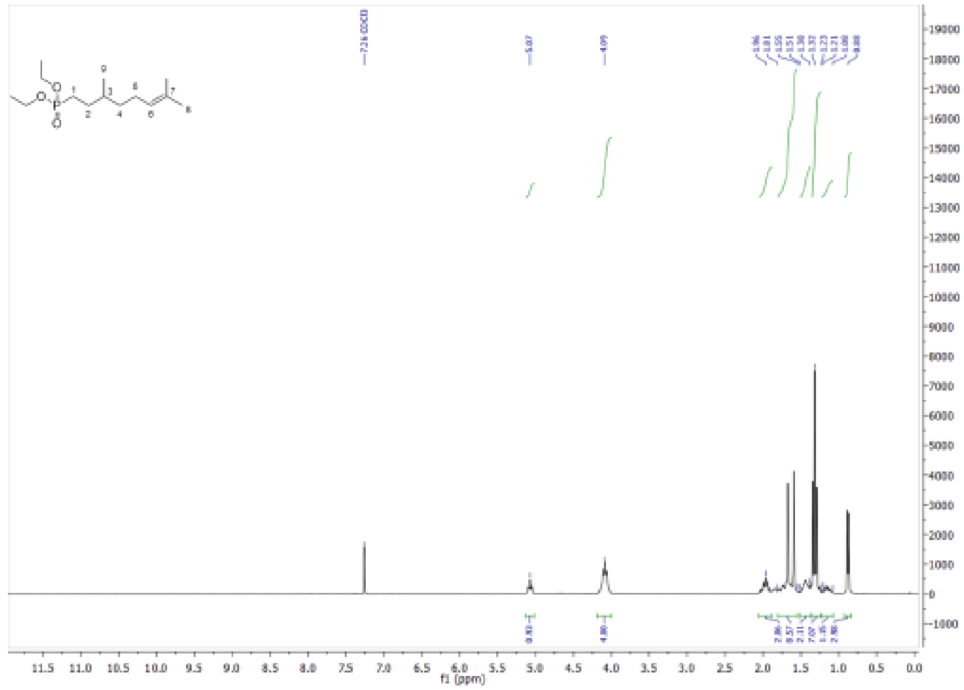


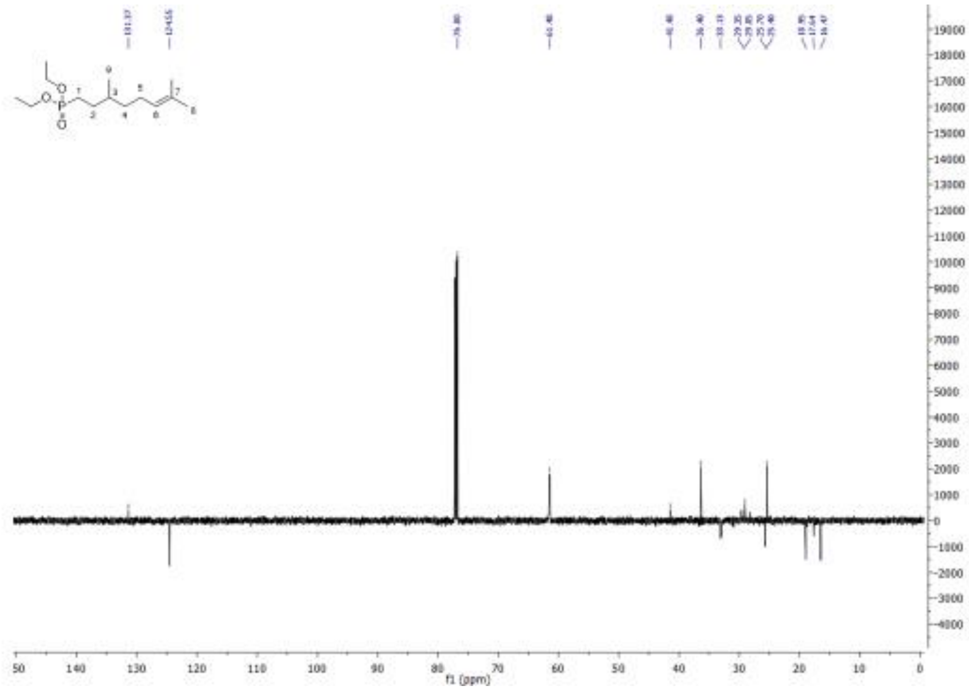

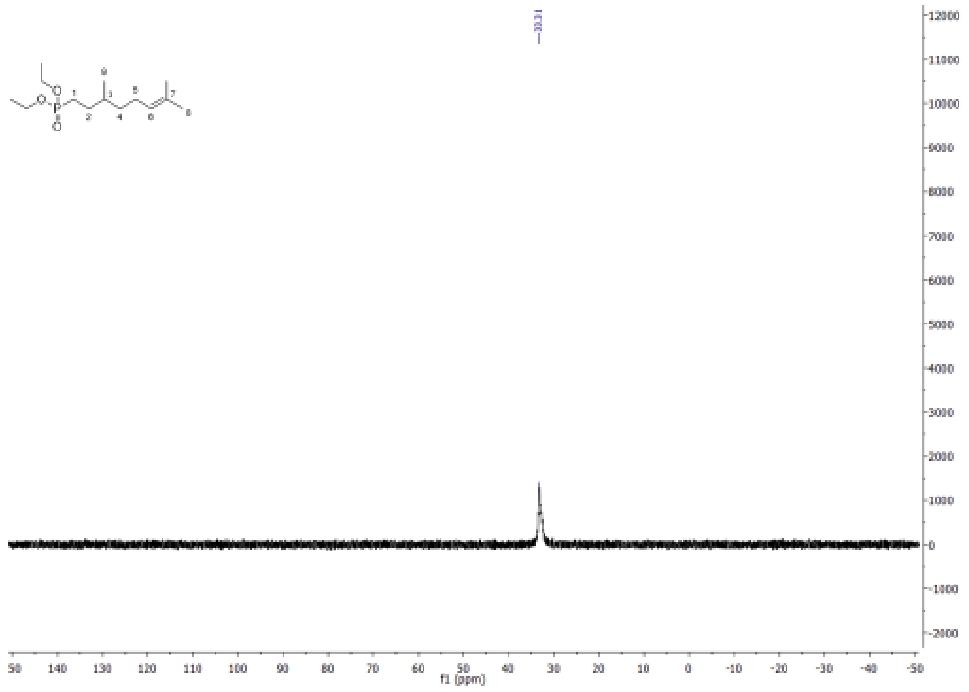
33.31
f1 (ppm)

**(3,7-Dimethyloct-6-en-1-yl)phosphonic acid 27**

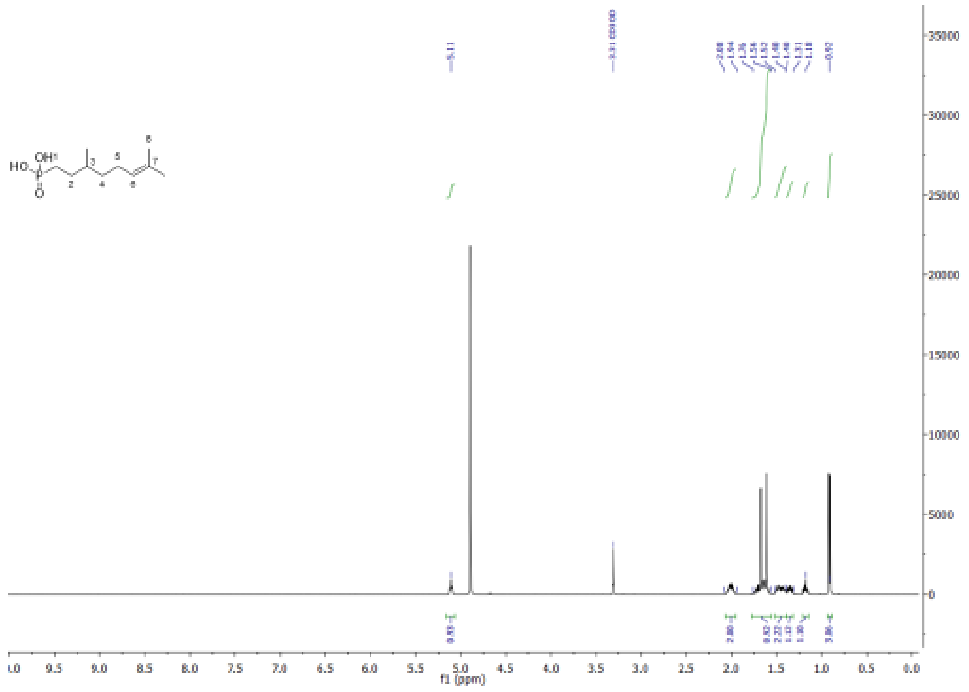

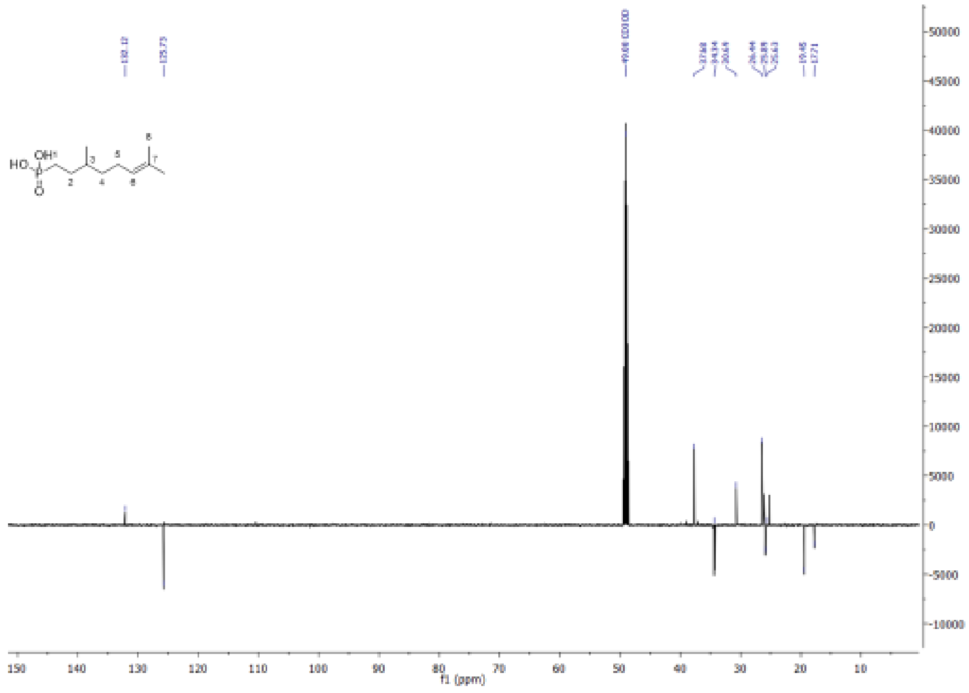

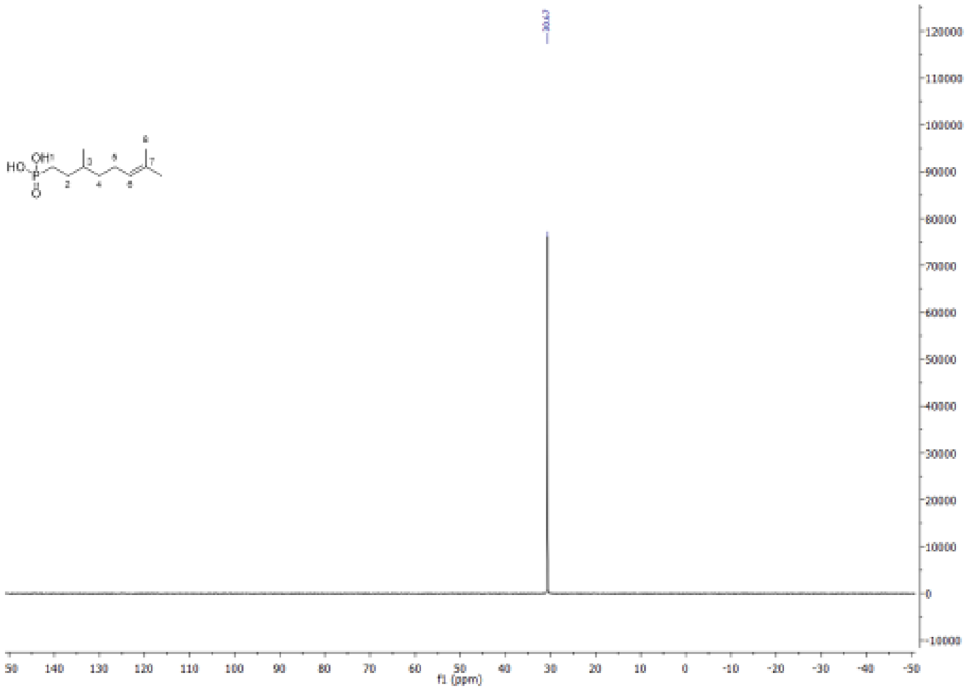

## Diethyl (3,7-dimethyloctyl)phosphonate

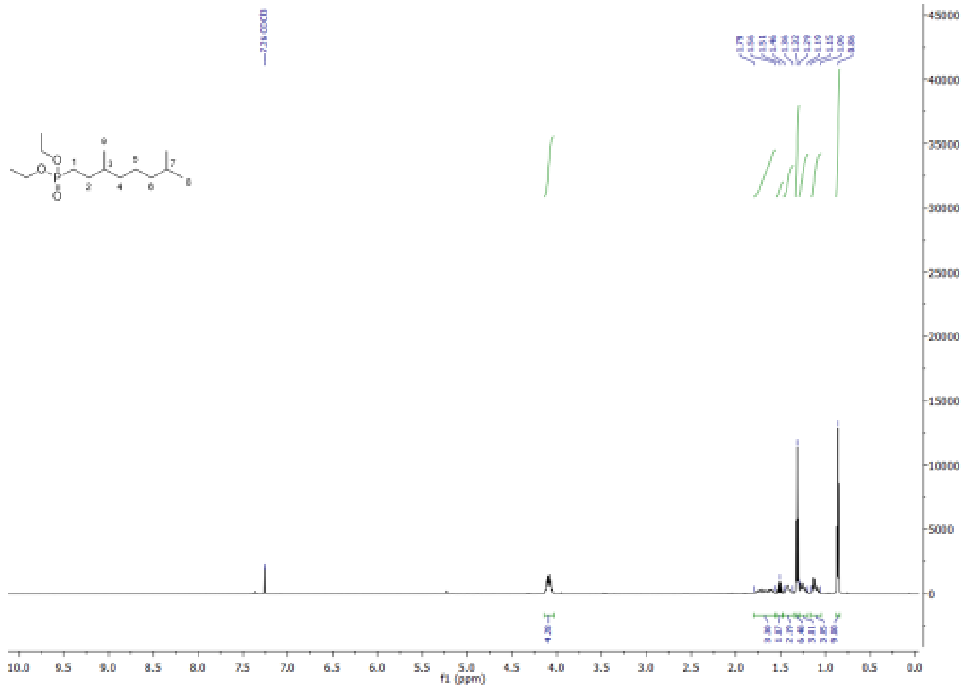


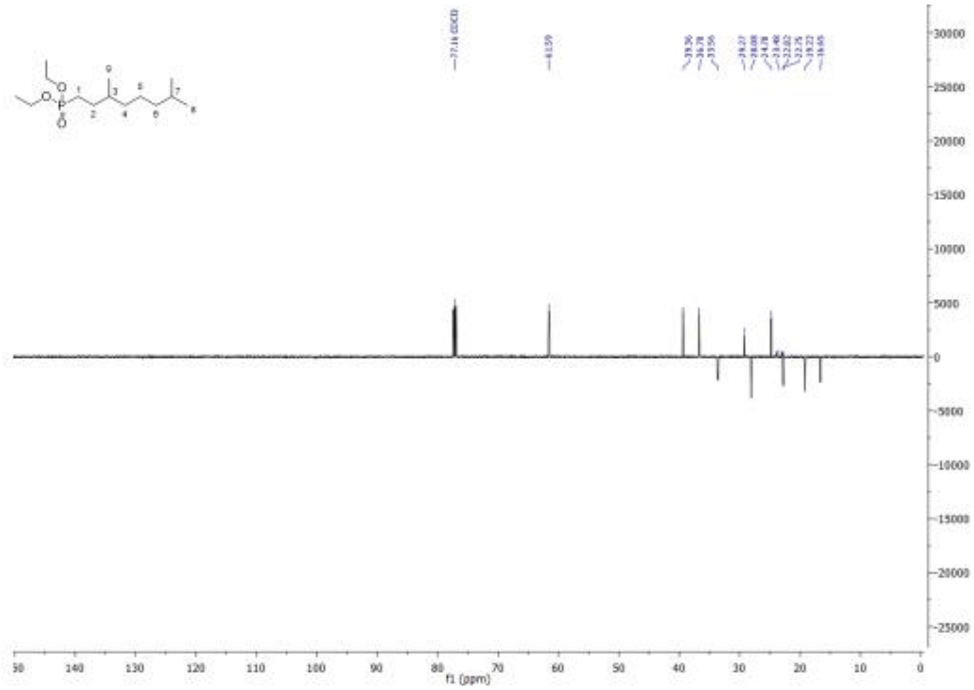

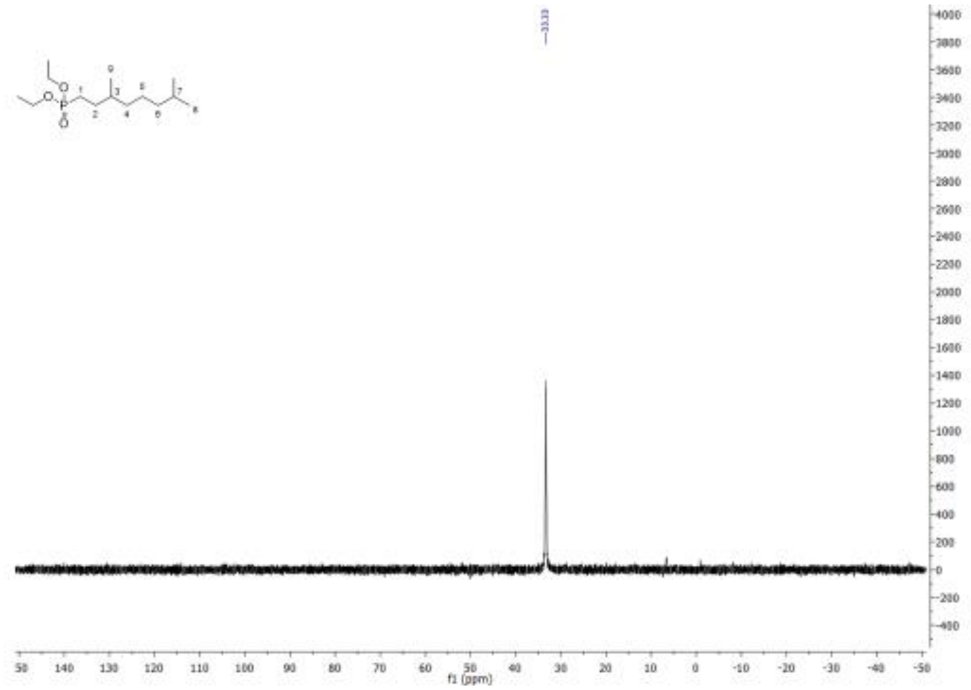

## (3,7-Dimethyloctyl)phosphonic acid 28

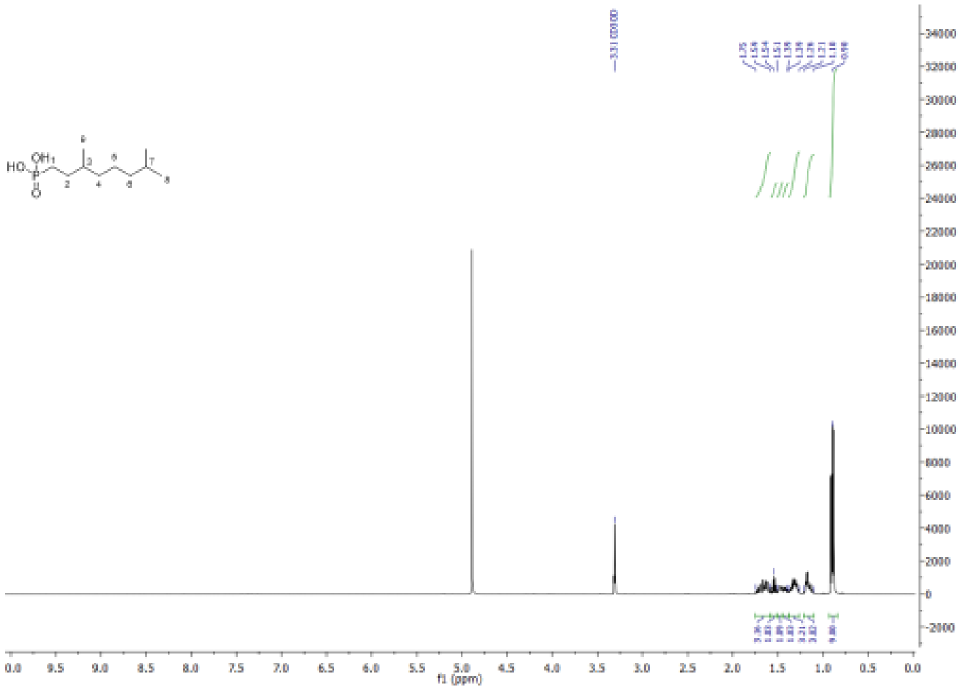


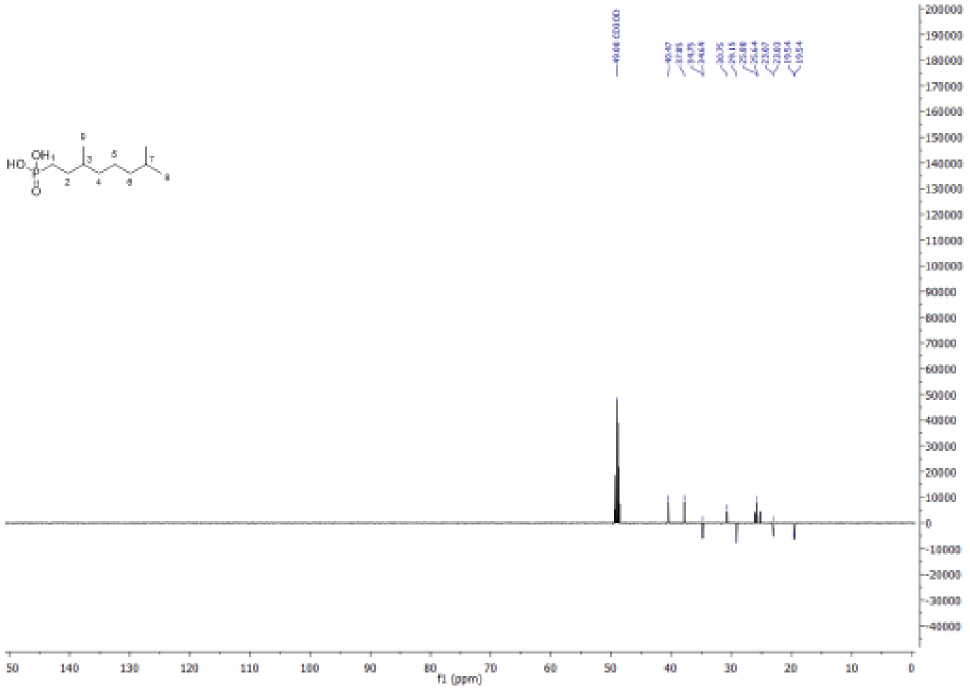

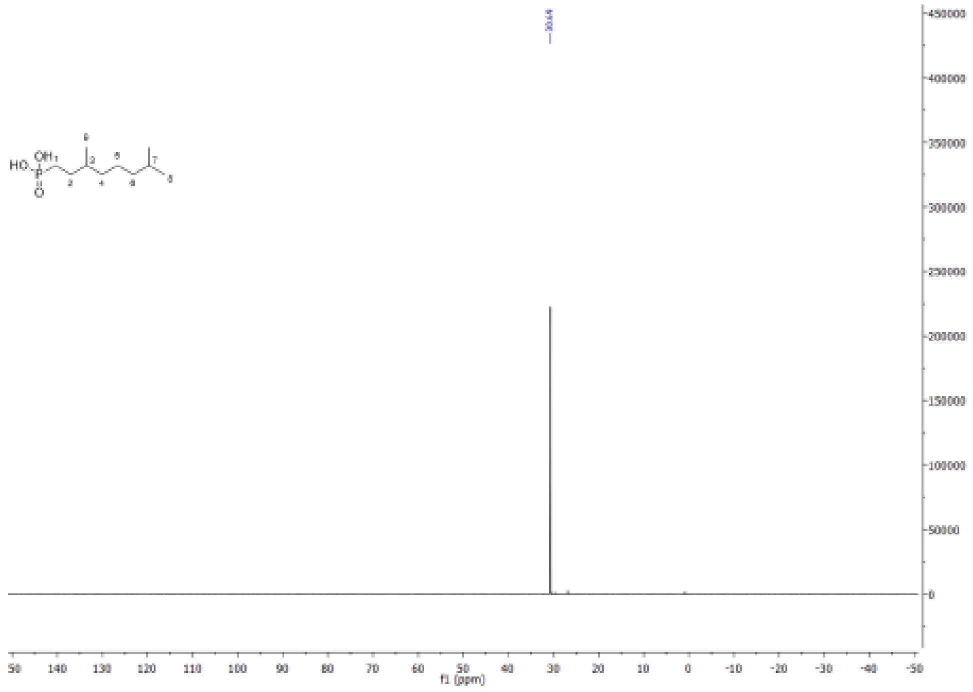

30.69
f1 (ppm)

# Chip filter test (Table S2)

Evaluation of the anti-corrosive properties of the substances for iron was carried out according to DIN 51360-02-A using the aqueous, TEA-neutralized solution of the acidic corrosion inhibitors. Grey cast iron turnings on filter paper were treated with test solutions for 2h at room temperature in either hardwater (20 dGH: $CaCl_2$ 340 mg/L, $MgSO_4$ 60.0 mg/L) or 0.5% aqueous NaCl (pH 8.3 ± 0.5). 1.5 eq TEA were added for each acidic proton in acidic corrosion inhibitors. As an example, carboxyphosphonic acid **3** contains 3 acidic protons and 4.5 eq TEA were thus added as alkaline additive. 2 mL of the appropriate test solution was incubated with 2.0 g of sieved grey cast iron turnings for 2 h, before rinsing and visual scoring according to DIN 51360-02-A.

**Table S2.** Selected results of the chip filter test according to DIN 51360-2.

| Entry | Acid | Mol. weight | Conc. [mmol/L] | Conc. [wt.%] | Score |
|---|---|---|---|---|---|
| 1 | **TC®** | 468.56 | 20 | 0.937 | 0 |
| 2 | **TC®** | 468.56 | 10 | 0.469 | 1 |
| 3 | **TC®** | 468.56 | 5 | 0.234 | 4 |
| 4 | **OPA** | 194.21 | 10 | 0.194 | 2 |
| 5 | **7** | 218.23 | 36 | 0.786 | 0 |
| 6 | **7** | 218.23 | 18 | 0.393 | 0 |
| 7 | **7** | 218.23 | 10 | 0.218 | 1 |
| 8 | **7** | 218.23 | 9 | 0.196 | 3 |
| 9 | **27** | 220.25 | 36 | 0.793 | 0 |
| 10 | **27** | 220.25 | 18 | 0.396 | 1 |
| 11 | **27** | 220.25 | 10 | 0.220 | 2 |
| 12 | **27** | 220.25 | 9 | 0.198 | 4 |
| 13 | **28** | 222.26 | 36 | 0.800 | 0 |
| 14 | **28** | 222.26 | 18 | 0.400 | 1 |
| 15 | **28** | 222.26 | 10 | 0.222 | 3 |
| 16 | **28** | 222.26 | 9 | 0.200 | 4 |
| 17 | **3** | 266.27 | 12.5 | 0.333 | 0 |
| 18 | **3** | 266.27 | 10 | 0.266 | 1 |
| 19 | **3** | 266.27 | 6.25 | 0.166 | 3 |
| 20 | **18** | 364.46 | 20 | 0.729 | 0 |
| 21 | **18** | 364.46 | 10 | 0.364 | 1 |
| 22 | **18** | 364.46 | 5 | 0.182 | 4 |
| 23 | **24** | 380.46 | 27.6 | 1.050 | 0 |
| 24 | **24** | 380.46 | 13.8 | 0.525 | 1 |
| 25 | **24** | 380.46 | 10 | 0.380 | 2 |
| 26 | **24** | 380.46 | 5 | 0.190 | 4 |
| 27 | **12** | 218.23 | 10 | 0.218 | 0 |
| 28 | **6** | 218.23 | 18 | 0.393 | 1 |

# Nyquist Plots

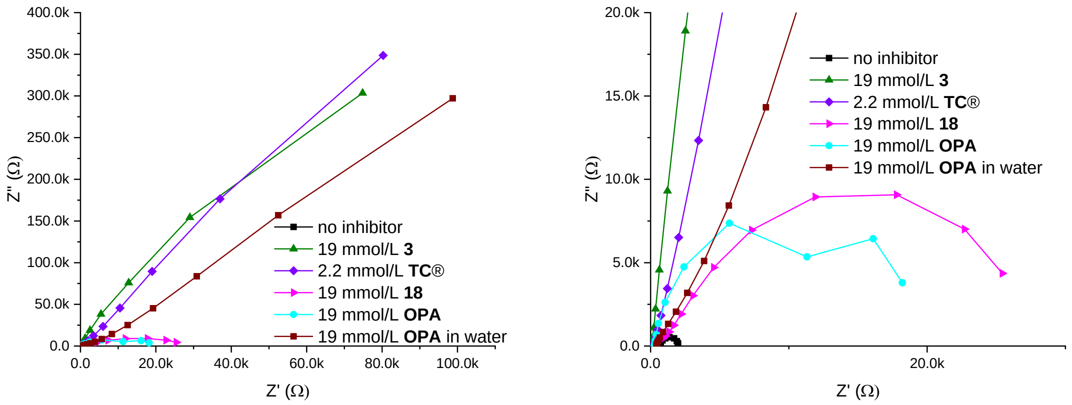


**Figure S1.** Nyquist plots of electrochemical impedance measurements of steel (SJR235) in in hard water (20dGH, pH 8.3 ± 0.5) and demineralized water (only for **OPA**, pH 8.5 adjusted with 3 eq TEA) with different corrosion inhibitors (neutralized with TEA).

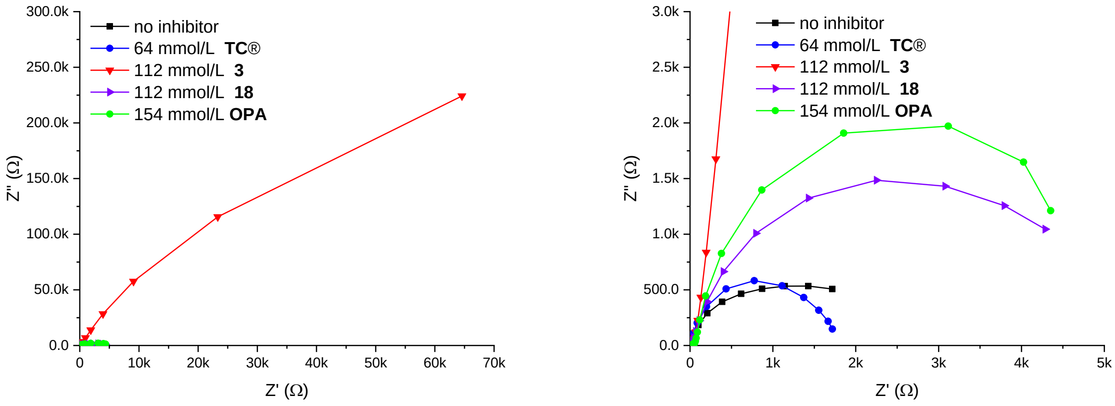


**Figure S2.** Nyquist plots of electrochemical impedance measurements of steel (SJR235) in aqueous 3.0% NaCl (pH 8.3 ± 0.5) with different corrosion inhibitors (neutralized with TEA).

# References


1. V. Chavane, *Compt. rend.*, 1947, **224**, 406-408.
2. L. Coudray, K. Bravo-Altamirano and J. L. Montchamp, *Org. Lett.*, 2008, **10**, 1123-1126.
3. H. Francois and R. Lalande, *Cr. Acad. Sci. C Chim.*, 1974, **279**, 117-119.
4. G. Biresaw and G. B. Bantchev, *Tribol. Lett.*, 2015, **60**, article number 11.
5. F. Millet, R. Auvergne, S. Caillol, G. David, A. Manseri and N. Pébère, *Prog. Org. Coat.*, 2014, **77**, 285-291.
6. J. C. Siu, J. B. Parry and S. Lin, *J. Am. Chem. Soc.*, 2019, **141**, 2825-2831.
